# N‐Acenoacenes

**DOI:** 10.1002/chem.201903646

**Published:** 2019-10-18

**Authors:** Lukas Ahrens, Sebastian Hahn, Frank Rominger, Jan Freudenberg, Uwe H. F. Bunz

**Affiliations:** ^1^ Organisch-Chemisches Institut Ruprecht-Karls-Universität Heidelberg Im Neuenheimer Feld 270 69120 Heidelberg, FRG Germany; ^2^ InnovationLab Speyerer Straße 4 69115 Heidelberg, FRG Germany; ^3^ Centre for Advanced Materials (CAM) Ruprecht-Karls-Universität Heidelberg Im Neuenheimer Feld 225 69120 Heidelberg, FRG Germany

**Keywords:** 2D acenes, aromaticity, azaacenes, electronic coupling, semiconductors

## Abstract

The syntheses of new, fourfold alkynylated tetraazaacenoacenes (tetraazaanthracenoanthracene, tetraazatetracenotetracene and tetraazapentacenopentacene) are reported. This novel heteroacenoacene motif exhibits surprisingly strong electronic coupling between its constituting diazaacene units.

## Graphical abstract

**Two are better than one**: Soluble, alkynylated azaacenoacenes were synthesized through Buchwald–Hartwig coupling of *ortho*‐diamines with bistriflated/nonaflated dibromonaphthalenes. The two azaacene units of this long‐forgotten bisacene motif exhibited surprisingly strong electronic coupling.

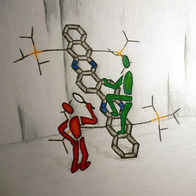

Acenes, N‐heteroacenes, and larger aromatic and N‐heteroaromatic species are important materials for charge transport and singlet fission.^[1]^ “True” acenes are defined by the presence of only one Clar sextet in the whole aromatic system of linearly annulated benzene rings. *Cata*‐condensation of two/four (hetero)aromatic rings onto an acene core leads to a situation, in which one or two rings in the larger aromatic system are chemically similar to the central ring of a triphenylene unit; reduced nucleus‐independent chemical shift (NICS) values and blueshifted absorption results. For tetrabenzoheptacene **A** ^[2]^ and heptacene **B** ^[3]^ notable differences arise, because **A** displays an absorption maximum similar to that of hexacene, blueshifted in comparison to that of heptacene **B** (Figure 1).

**Figure 1:**
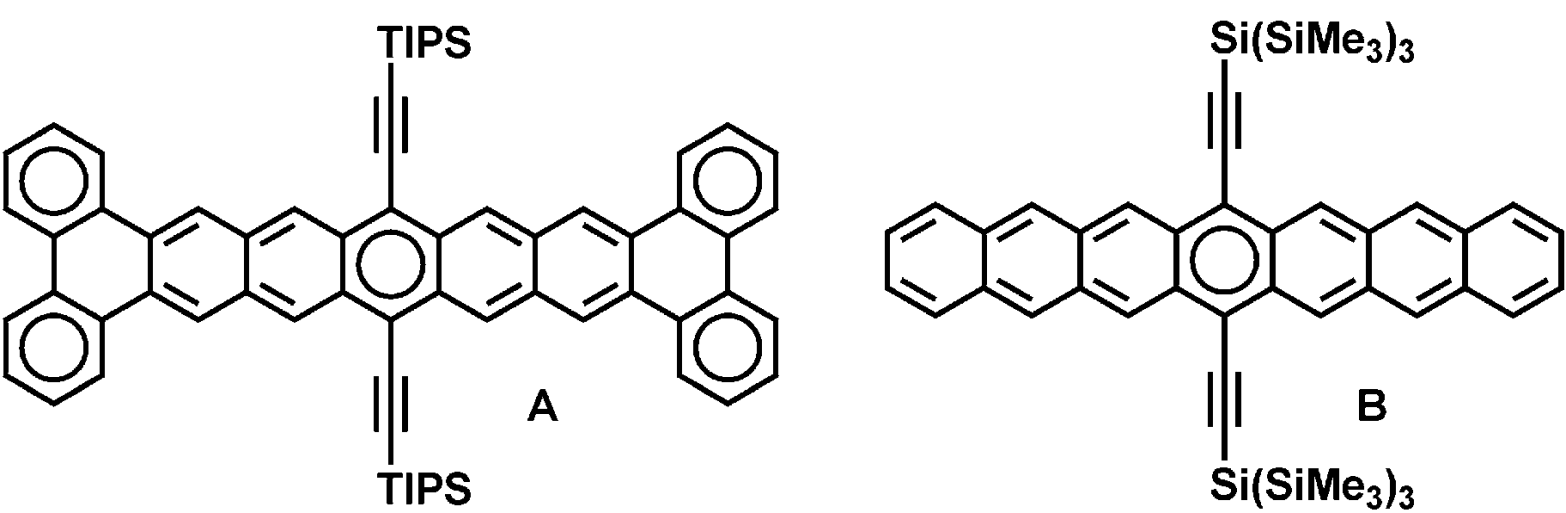
Chemical structures of tetrabenzoheptacene **A** and heptacene **B**.[^2^, ^3^]

This effect is even more pronounced for acenes connected to, or held together by, other aromatic moieties, for example, central pyrene or coronene units. In these cases, the acene subunits are mostly electronically decoupled.[^4^, ^5^, ^6^, ^7^, ^8^, ^9^] Here, we demonstrate that stitching together two azaacenes at their 1,2‐bond^[10]^ results in species for which absorption and emission are redshifted when compared to that of their monomeric parents.

The targets **3 a**–**c** are accessed with surprising ease, it must be noted that this structural motif is unknown for the larger hydrocarbon analogues of the so called 2D‐acenes; anthracenoanthracene was synthesized by Clar as early as 1929.^[11]^ Starting from **1 a** ^[12]^ or **1 b** (Supporting Information) Buchwald–Hartwig coupling[^13^, ^14^] with **2 a**–**c** gives well‐soluble **3 a**–**c** in yields from 9–51 % (Scheme 1). Compound **1 b** is slightly superior here, probably due to its increased stability;^[15]^ the triflates give the targets in 8–48 % after column chromatography followed by repeated preparative gel permeation chromatography. RuPhos Pd G1^[16]^ performs best; other catalyst systems (see Supporting Information) or longer reaction times are less effective. It must be noted that the quantity of **3 c** obtained was sufficient for optical characterization and analysis by proton NMR, mass spectrometry and infrared spectroscopy, but it did not suffice for characterization by cyclic voltammetry or carbon NMR—several reruns of the reaction yielded only trace amounts. Thus, we do not fully include pentacenopentacene **3 c** into our discussion, although the present study suggests that it can be accessed and the species stable enough for characterization.

**Scheme 1:**
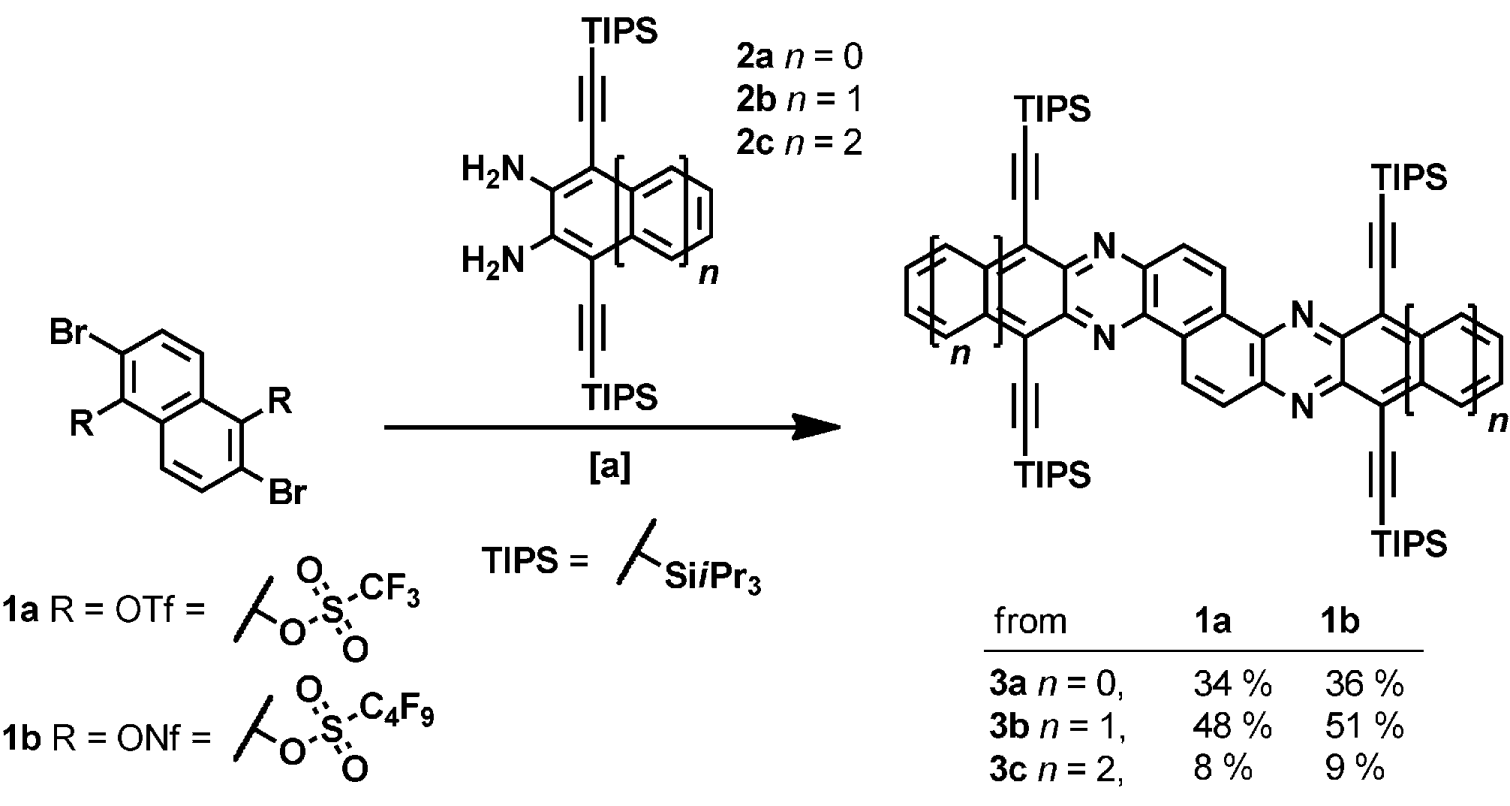
Buchwald–Hartwig coupling of the N‐acenoacenes **3 a**–**c** by using diamines **2 a**–**c** and triflates **1 a**,**b**. [a] RuPhos Pd G1 (10 mol %), cesium carbonate (6.0 equiv), toluene, 110 °C, 24 h.

Figure 2 displays photographs of solutions of **3 a**–**c** in CH_2_Cl_2_ under daylight and UV‐light irradiation. Remarkable is the strong fluorescence of **3 b**, which overwhelms the true color (green) of the solution even in daylight (see Supporting Information).

**Figure 2:**
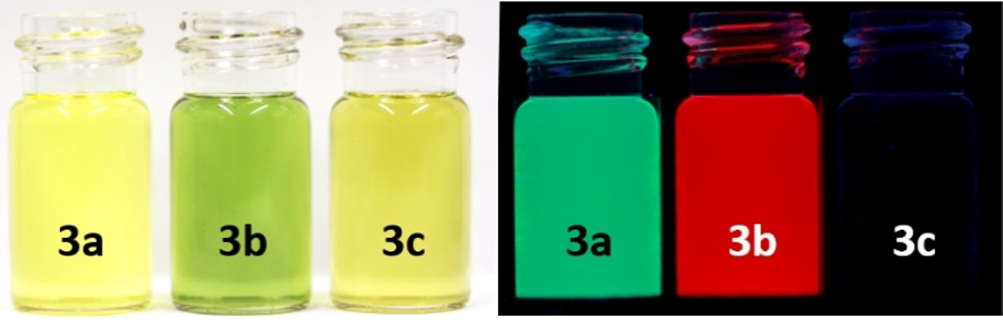
Photographs of **3 a**–**c** (from left to right) under daylight (left) and UV light (right, *λ*_ex_=365 nm) in CH_2_Cl_2_.

Figure 3 (top) and Table 1 depict the optical properties of **3 a**–**c** in CH_2_Cl_2_. Upon increasing the size of the acene subunits the absorption maximum redshifts from 480 to 760 nm. The bisanthracene **3 a** is hardly emissive (*λ*_max, em_=505 nm, *Φ*=9 %) and the bispentacene **3 c** is nonfluorescent in solution, whereas surprisingly bistetracene emits strongly (*λ*_max, em_=657 nm, *Φ*=38 %). Compounds **3 a** and **3 b** are photostable in degassed solution: After irradiation for 2.5 h with a hand‐held UV lamp in CH_2_Cl_2_ at 365 nm, both their absorbances were retained (see Supporting Information).

**Figure 3:**
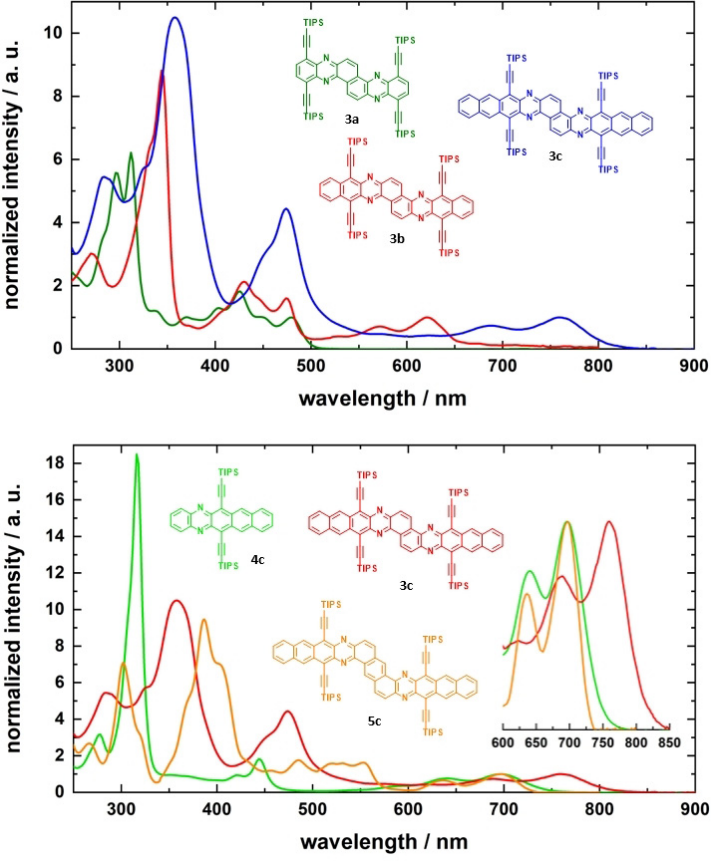
Top: Normalized absorption spectra of **3 a**–**c** in CH_2_Cl_2_. Bottom: **3 c**, **4 c** and **5 c** in CH_2_Cl_2_.

**Table 1:** Optical properties of **3 a**–**c**, **4 a**–**c** and **5 a**–**c** in CH_2_Cl_2_.

Compound	*λ*_max, abs_ [nm]	*λ*_onset, abs_ [nm]	*λ*_max, em_ [nm]	Stokes shift [cm^‐1^]	*ϵ* [×10^4^ m ^−1^ cm^−1^]^[a]^	*Φ*	*τ* [ns]
**3 a**	480	500	505	1031	9.83	0.09	15.7
**4 a**	438	468	487	2297	–	0.02^[b]^	0.37^[b]^
**5 a** ^[c]^	480	495	491	467	5.15	0.24	–
**3 b**	622	657	657	856	14.34	0.38	1.27
**4 b**	570	604	608	1096	–	–	–
**5 b** ^[c]^	575	599	597	641	–	–	–
**3 c**	760	818	–	–	–	–	–
**4 c**	696	750	739	836	–	–	–
**5 c** ^[c]^	698	729	716	360	6.79	–	–

[a] At *λ*_max, abs_. [b] Taken from Ref. ^[17a]^. [c] Taken from Ref. ^[18]^.

To evaluate the electronic interaction of the acene units in **3 a**–**c**, we compared the latter compounds to **4 a**–**c** ^[17]^ and **5 a**–**c** ^[18]^ (Figure 4), which have the same number of linearly annulated rings (Figure 3, bottom). The absorption maximum of diazapentacene **4 c** is redshifted by 2 nm compared to that of heterophene **5 c**; pentacenopentacene **3 c** absorbs at significantly lower energy (Δ*λ*_max, abs_=64 nm, Δ*ν*=1210 cm^−1^). This is also true for the tetracene series. Anthracenes **3 a** and **5 a**, however, have the same absorption maximum, but both are redshifted compared to the absorption spectrum of phenazine **4 a**. Thus, junction at the 1,2‐bond of two diazaacenes shows the largest electronic interactions for *n*>0, when compared to other topologies.^[19]^ This is mirrored by the first reduction potentials; **4 b** is electrochemically reduced at −1.23 V, **5 b** at −1.20 V, but **3 b** is reduced at −1.03 V suggesting a 0.2 eV strong coupling between the azaacene units.

**Figure 4:**
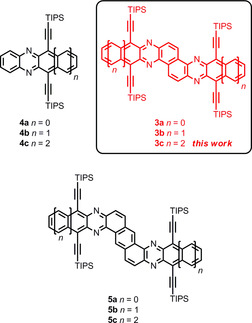
Acenes **4 a**–**c**,^[17]^ N‐acenoacenes **3 a**–**c**, and N‐heterophenes **5 a**–**c**.^[18]^

The same trend is found for the calculated LUMO levels (Table 2), which are lowest for the acenoacene series (*n*>0). The HOMO energies are virtually identical for **3/4/5 a**,**b**,**c**; the different topologies are therefore a method to fine‐tune the electron affinities.

**Table 2:** Experimental and calculated (gas‐phase) properties of **3 a**–**c**, **4 a**–**c** and **5 a**–**c**.

Compound	*E*_(0/−)_ [V]^[a]^	Ionization potential/HOMO [eV] exptl^[d]^/calcd^[e]^	Electron affinity/LUMO [eV] exptl^[b]^/calcd^[e]^	Gap [eV] exptl^[c]^/calcd^[e]^
**3 a**	−1.26	−6.32/−6.01	−3.84/−3.35	2.48/2.66
**4 a** ^[f]^	−1.68	−6.12/−5.97	−3.42/−3.08	2.70/2.88
**5 a** ^[f]^	−1.42	−6.18/−5.97	−3.68/−3.20	2.50/2.77
**3 b**	−1.03	−5.96/−5.53	−4.07/−3.59	1.89/1.94
**4 b** ^[f]^	−1.23	−5.99/−5.54	−3.87/−3.35	2.12/2.20
**5 b** ^[f]^	−1.20	−5.97/−5.56	−3.90/−3.44	2.07/2.12
**3 c**	–	–/−5.24	–/−3.73	1.52/1.51
**4 c** ^[f]^	−1.05	−5.80/−5.25	−4.05/−3.50	1.75/1.75
**5 c** ^[f]^	−1.42	−5.38/−5.21	−3.68/−3.52	1.70/1.69

[a] First‐reduction potentials measured by cyclic voltammetry (CV) in tetrahydrofuran at room temperature with Bu_4_NPF_6_ as the electrolyte against Fc/Fc^+^ as an internal standard (−5.10 eV) at 0.2 V s^−1^.^[20]^ [b] Electron affinity_exptl_ = −e×(5.10 V + *E*_Red._). [c] gap_exptl_ calculated from *λ*_onset_ in CH_2_Cl_2_. [d] HOMO_exptl_ = LUMO_exptl_ − gap_exptl_. [e] Obtained from DFT calculations (TURBOMOLE B3LYP/def2‐TZVP//Gaussian 09 B3LYP/6‐311++G**; TMS groups were used instead of TIPS). [f] Data for **4 a**–**c** were taken from Ref. ^[17]^ and for **5 a**–**c** (hot pyridine) from Ref. ^[18]^.

Compounds **4 a**–**c** (Figure 5) display the NICS(1)‐values expected for acenes, the aromatic rings introducing kinks to **5 a**–**c** display small NICS values, similar to phenanthrene,^[21]^ suggesting that they do not fully contribute to the constituent acene unit. The central naphthalene connectors of acenoacenes **3 a**–**c**, however, are more aromatic, explaining the lower bandgaps and the higher degree of interaction of the acene subunits.

**Figure 5:**
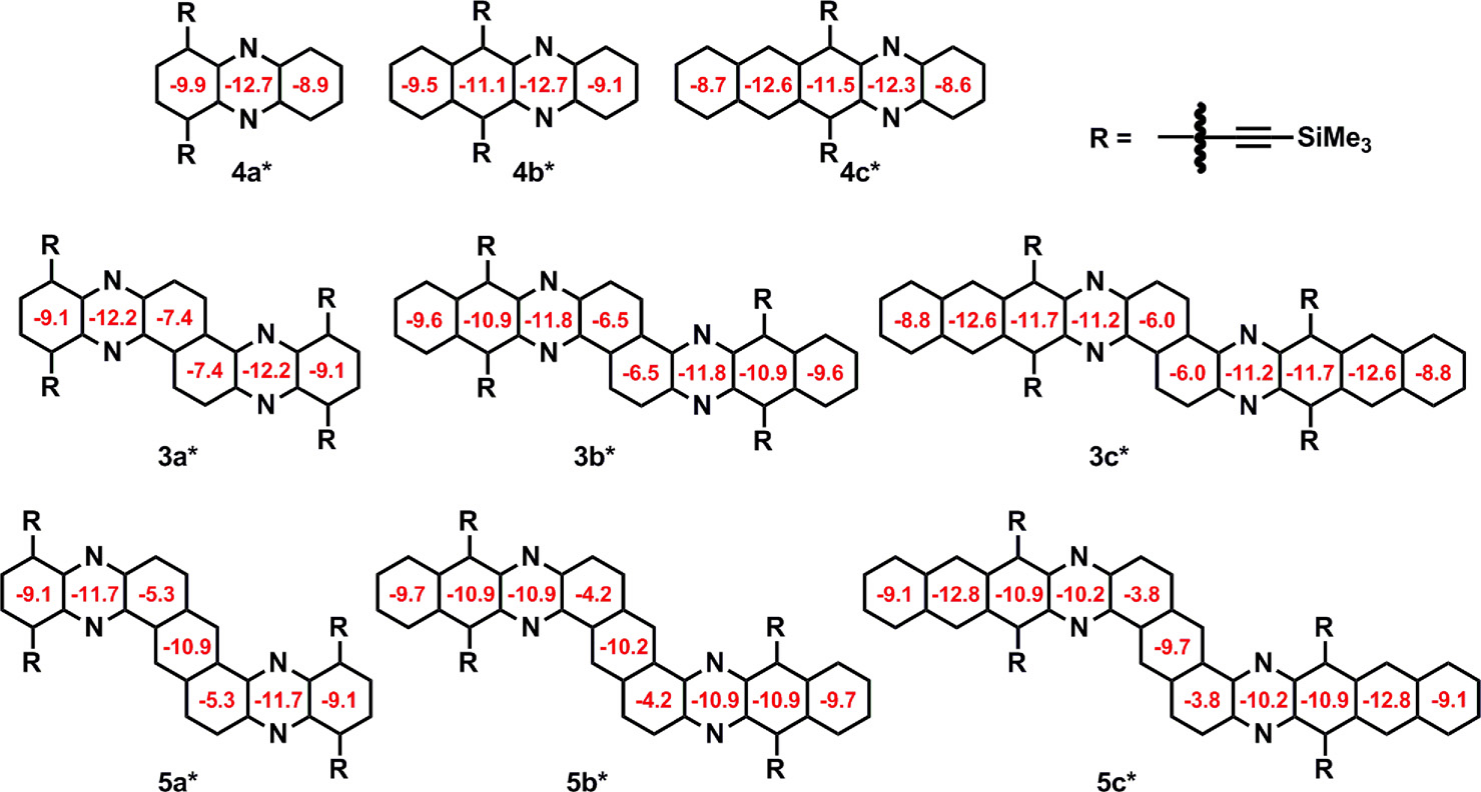
NICS(1) values of compounds **3 a**–**c**, **4 a**–**c**, and **5 a**–**c** (B3LYP/6‐311++G** level); TMS groups were used instead of TIPS.

Single‐crystalline specimen of **3 a**,**b** suitable for X‐ray analysis were obtained through crystallization from chloroform. The bond lengths and bond angles are in excellent agreement with the expected and calculated values. Both solid‐state structures do not show appreciable π–π interactions and are instead governed by close contacts of the four TIPS groups to the neighboring molecules, thereby insulating the π‐systems from each other (Figure 6).

**Figure 6:**
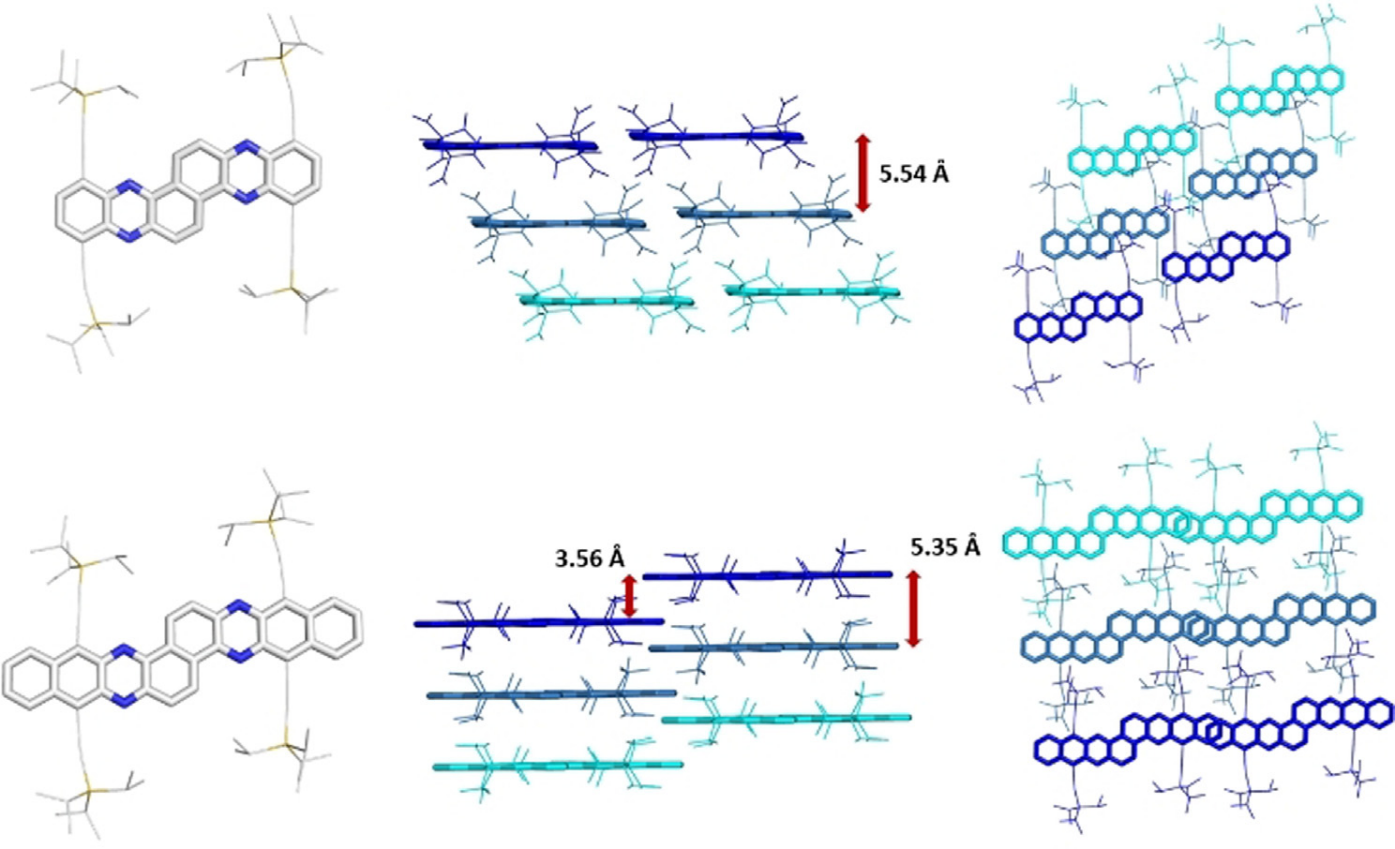
Solid‐state structures of **3 a** and **3 b**; distances between neighboring molecules; visualization of overlap and packing.

In conclusion, joining two N‐heteroacene units through their 1,2‐bonds leads to targets in which two acenes are joined by a common aromatic bond; their interaction is increased in comparison to other modes of connecting acene nuclei. One can look at **3 a**–**c** as species in which naphthalene is the central core to which further acene units are added in the 1,2 and 5,6‐positions, whereas in **5 a**–**c** an anthracene nucleus plays the same role. If a central benzene ring connects the acene units as in **5 a–c**,^[19]^ the electronic interactions of the acene units are not as pronounced as in the acenoacene case. The latter and their N‐heterocyclic congeners play a privileged role as the interaction of their constituent (hetero)acene parts is maximized.

## Experimental Section

### Syntheses

**1 a**,^[12]^ **2 a**,^[22]^ **2 b**,^[17b]^ **2 c**[^17c^, ^22^] and **4 a**–**c** ^[17]^ were synthesized according to literature procedure.

### Crystallographic data

CCDC 1946373 (**3a**) and 1946372 (**3b**) contain the supplementary crystallographic data for this paper. These data are provided free of charge by The Cambridge Crystallographic Data Centre.

### General procedure for the preparation of N‐acenoacenes

In a heat‐gun‐dried Schlenk tube, under an atmosphere of argon, was added **1** (1.0 equiv), the *ortho*‐diamine **2 a**–**c** (2.0 equiv), cesium carbonate (6.0 equiv) and RuPhos Pd G1 (10 mol %). Then, anhydrous, degassed toluene was added, and the reaction mixture stirred at 110 °C for 24 h. The mixture was cooled to room temperature and diluted with water (10 mL). The phases were separated, and the aqueous layer was extracted with dichloromethane (3×10 mL). The combined organic phases were washed with brine (10 mL), dried over magnesium sulfate and filtrated. The solvent was removed under reduced pressure and the crude product was absorbed on Celite. After flash column chromatography [petroleum ether/diethyl ether 250:1 (*v*/*v*)] and gel permeation chromatography (toluene) the product was isolated.

## Conflict of interest

The authors declare no conflict of interest.

## Supporting information

As a service to our authors and readers, this journal provides supporting information supplied by the authors. Such materials are peer reviewed and may be re‐organized for online delivery, but are not copy‐edited or typeset. Technical support issues arising from supporting information (other than missing files) should be addressed to the authors.

Supplementary
