# Supplementary material for: N‐Acenoacenes
Source: Chemistry. 2019 Oct 18;25(64):14522–6. doi: 10.1002/chem.201903646 (PMC7687221; doi:10.1002/chem.201903646)
Supplement: Supplementary file 1 — Supplementary [file CHEM-25-14522-s001.pdf]

CHEMISTRY
A European Journal

Supporting Information

N-Acenoacenes

Lukas Ahrens,[a] Sebastian Hahn,[a] Frank Rominger,[a] Jan Freudenberg,[a, b] and Uwe H. F. Bunz*[a, c]

chem_201903646_sm_miscellaneous_information.pdf

Sections

S1. General remarks..S3
S2. Synthesis……...S6
S3. Catalyst screening……………………………………………………………………...S10
S4. UV-Vis spectra..S11
S5. UV-Vis absorption stability study...S12
S6. Electrochemistry/cyclovoltammetry...S14
S7. Quantum-chemical calculations of the FMOs...S15
S8. NMR spectra…..S17
S9. X-ray single-crystal structure analysis..S26

S1. General Remarks

Reagents and solvents for synthesis

All reagents were obtained from commercial suppliers and were used without further purification if not otherwise stated. Deuterated solvents were purchased from Sigma-Aldrich Laborchemikalien GmbH (Seelze, Germany). All reactions requiring exclusion of oxygen and moisture were carried out in heat-gun dried glassware under a dry and oxygen free nitrogen or argon atmosphere using Schlenk and glovebox techniques.

Column chromatography

Column chromatography was performed using silica gel from Macherey, Nagel & Co. KG (Düren, Germany) (particle size: 0.040 - 0.063 mm). For TLC Polygram Sil G/UV 254 plates from Macherey, Nagel & Co. KG (Düren, Germany) were used and examined under UV-light irradiation (254 nm and 365 nm). Before column chromatography the crude product was mixed with Celite 545 and DCM to make a slurry. The solvent was removed by rotary evaporation to get a dry powder.

Gel permeation chromatography

Preparative GPC was performed on Bio-Beads® (S-X1 Beads, 200 - 400 Mesh, crosslinked polystyrene), purchased from Bio-Rad Laboratories, Inc.

^{1}H-NMR spectra and ^{13}C-NMR spectra

All spectra were recorded at room temperature on a Bruker Avance III 600 (^{1}H: 600 MHz, ^{13}C: 151 MHz) or Bruker Avance III 500 (^{1}H: 500 MHz, ^{13}C: 126 MHz). ^{13}C-NMR spectra were measured proton decoupled if not stated otherwise. NMR spectra were integrated and processed using the Software TopSpin 3.5pl5 (Bruker). For calibration the residual solvent peaks were referenced.[1] Chemical shifts δ are reported in ppm and coupling constants J in Hz. The following abbreviations describe the observed multiplicities: s = singlet, d = doublet, m = multiplet. *Via* 2D NMR methods, COSY, HSQC(me) and HMBC, and supported by DFT calculations (B3LYP/6–311++G** level) assignments of proton signals were performed.

IR spectra

All spectra were recorded neat at room temperature on a Jasco FT/IR-4100. Signals are reported in wavenumbers [cm^{-1}].

Mass spectrometry

The mass spectra were recorded using the following instruments: Bruker ApexQe hybrid 9.4 T FT-ICR (MALDI, ESI); Bruker AutoFlex Speed time-of-flight (MALDI).

Melting points

Melting points were determined in open glass capillaries with a Melting Point Apparatus MEL-TEMP (Electrothermal, Rochford, UK).

X-ray single-crystal structure analysis

X-ray single-crystal structure analyses were measured on a Bruker Smart APEX-II Quazar Area Detector diffractometer by Dr. F. Rominger (Heidelberg University). Diffraction intensities were corrected for Lorentz and polarization effects. An empirical absorption correction was applied using SADABS based on the Laue symmetry of reciprocal space. Heavy atom diffractions were solved by direct methods and refined against F2 with the full matrix least square algorithm. Hydrogen atoms were either isotropically refined or calculated. The structures were solved and refined using the SHELXTL software package.

UV-Vis and fluorescence spectroscopy

Absorption spectra were recorded on a Jasco UV-Vis V-660 or Jasco UV-Vis V-670. Fluorescence spectra were recorded on a Jasco FP-6500.

Quantum yields

Quantum yields were determined by an Ulbricht sphere (6 inch) using a PTI QuantaMaster 40 equipped with a Hamamatsu R928P Photomultiplier.

Quantum lifetimes

Quantum lifetimes were determined on a Horiba FluoroCube-01-NL lifetime spectrofluorometer with emission monochromator (Seya-Namioka type, 200 nm to 800 nm) and diode excitation (Nano-LED N-375L, 375 ± 10 nm, < 200 ps).

Camera

The photos were taken by a Canon EOS 7D under daylight or UV-light irradiation (λ = 365 nm).

Computational studies

Computational studies were carried out using DFT calculations on Turbomole 6.3.1 and Gaussian16. Geometry optimizations were performed using the B3LYP functional and def2-TZVP basis set. At this geometry, the absolute energy, NICS(1) values and FMO energies were assigned by a single-point approach at the B3LYP/6-311++G** level.[2]S2.

S2. Synthesis

2,6-Dibromonaphthalene-1,5-diyl bis(trifluoromethanesulfonate) (**1a**),[3] 3,6-bis((tri*iso*propylsilyl)ethynyl)benzene-1,2-diamine (**2a**),[4] 1,4-bis((tri*iso*propylsilyl)ethynyl)naphthalene-2,3-diamine (**2b**),[5b] 1,4-bis((tri*iso*propylsilyl)ethynyl)anthracene-2,3-diamine (**2c**),[6] 1,4-bis-

((tri*iso*propylsilyl)ethynyl)phenazine (**4a**),[5] 6,11-bis((tri*iso*propylsilyl)ethynyl)benzo[*b*]-phenazine (**4b**)[5] and 6,13-bis((tri*iso*propylsilyl)ethynyl)naphtho[2,3-*b*]phenazine (**4c**)[5] were synthesized according to literature procedures.

General procedure:

In a heatgun dried Schlenk tube under an atmosphere of argon was added **1** (1.00 equiv.), the *ortho*-diamine **2** (2.00 equiv.), caesium carbonate (6.00 equiv.) and RuPhos Pd G1 (10 mol%). Then anhydrous, degassed toluene was added, and the reaction mixture stirred at 110 °C for 24 h. The mixture was cooled to room temperature and diluted with water (10mL). The phases were separated, and the aqueous layer was extracted with dichloromethane (3×10 mL). The combined organic phases were washed with brine (10 mL), dried over magnesium sulfate and filtrated. The solvent was removed under reduced pressure and the crude product was absorbed on Celite®. After flash column chromatography (petroleum ether/diethyl ether 250:1 v/v) and gel permeation chromatography (toluene) the product was isolated.

2,6-Dibromonaphthalene-1,5-diyl bis(1,1,2,2,3,3,4,4,4-nonafluorobutane-1-sulfonate) (**1b**)

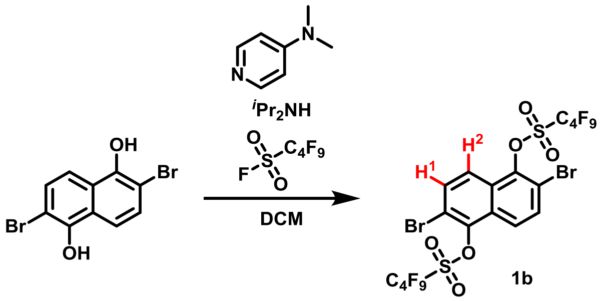

In a heatgun dried Schlenk tube under an atmosphere of argon was dissolved 2,6-dibromonaphthalene-1,5-diol (200 mg, 629 µmol, 1.00 equiv.), *N*,*N*-dimethylpyridin-4-amine (7.68 mg, 62.9 µmol, 0.10 equiv.) and di*iso*propylamine (153 mg, 212 µL, 1.51 mmol, 2.40 equiv.) in 10 mL anhydrous dichloromethane and the mixture cooled to 0 °C.

Nonafluorobutanesulfonic acid fluoride (440 mg, 262 µL, 1.38 mmol, 2.20 equiv.) was added dropwise, and the reaction mixture stirred at room temperature for 48 h. The mixture was poured in water (20 mL), the phases were separated, and the aqueous layer was extracted with dichloromethane (3×20 mL). The combined organic phases were washed with brine (10 mL), dried over magnesium sulfate and filtrated. The solvent was removed under reduced pressure and the crude product was absorbed on Celite®. After flash column chromatography

(petroleum ether/ethyl acetate 100:1 v/v) the product **1b** was isolated as a colorless solid (466 mg, 528 μmol, 84 %).

R_f = 0.80 (SiO_2; petroleum ether/ethyl acetate 10:1 v/v). Mp: 108 °C. ^{1}H-NMR ($CDCl_3$, 600.24 MHz, RT): δ = 8.04 (d, J = 9.05 Hz, 2H, H^2), 7.88 (d, J = 9.05 Hz, 2H, H^1) ppm. ^{13}C-NMR ($CDCl_3$, 150.93 MHz, RT): δ = 142.5, 133.3, 128.8, 123.2, 118.1, 116.9, 116.2, 115.0, 110.1 ppm. IR (ATR): $\tilde{\nu}$ = 3089, 1586, 1413, 1408, 1194, 1171, 1140, 1028, 937, 858, 815, 731, 687, 579, 497 cm^{-1}. HRMS (ESI$^+$) m/z: [M-H]$^+$: calcd. for $[C_{18}H_3{}^{79}Br^{81}BrF_{18}O_6S_2]^+$: 880.7424; found 880.7459; correct isotope distribution.

1,4,9,12-Tetrakis((tri*iso*propylsilyl)ethynyl)phenazino[2,1-*a*]phenazine (**3a**)

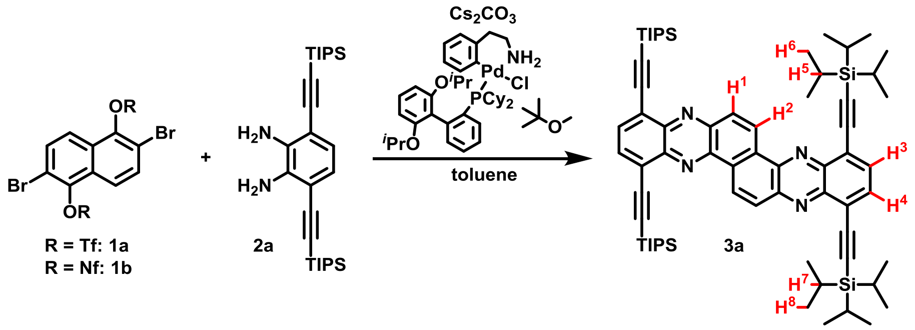

The **GP** was applied to **1a** (50.0 mg, 85.9 μmol, 1.00 equiv.) and **2a** (80.6 mg, 172 μmol, 2.00 equiv.) in 1 mL anhydrous, degassed toluene. Flash column chromatography (SiO_2; petroleum ether/diethyl ether 250:1 v/v) and gel permeation chromatography (toluene) yielded a yellow solid **3a** (31.1 mg, 29.5 μmol, 34%).

Applying the **GP** to **1b** (150 mg, 170 μmol, 1.00 equiv.) and **2a** (159 mg, 340 μmol, 2.00 equiv.) in 2 mL anhydrous, degassed toluene, flash column chromatography (SiO_2; petroleum ether/diethyl ether 250:1 v/v) and gel permeation chromatography (toluene) yielded a yellow solid **3a** (64.8 mg, 61.5 μmol, 36%).

R_f = 0.85 (SiO_2; petroleum ether/dichloromethane 2:1, v/v). Mp: ≥326 °C (decomposition). ^{1}H-NMR ($CDCl_3$, 600.24 MHz, RT): δ = 9.92 (d, J = 9.26 Hz, 2H, H^2), 8.39 (d, J = 9.26 Hz, 2H, H^1), 8.03 - 8.07 (m, 4H, H^3/H^4), 1.32 - 1.36 (m, 42H, H^5/H^6), 1.28 - 1.31 (m, 42H, H^7/H^8) ppm. ^{13}C-NMR ($CDCl_3$, 150.93 MHz, RT): δ = 144.5, 143.8, 142.5, 141.1, 134.4, 134.3, 132.3, 129.1, 129.0, 124.7, 124.6, 103.7, 103.6, 101.3, 100.6, 19.0, 19.0, 11.73, 11.7 ppm. IR (ATR): $\tilde{\nu}$ = 2941, 2862, 2358, 2147, 1461, 1354, 1239, 995, 881, 849, 793, 677, 661, 627, 589, 582, 460, 415 cm^{-1}. HRMS (MALDI$^+$) m/z: [M]$^+$: calcd. for $[C_{66}H_{92}N_4Si_4]^+$: 1052.6405; found 1052.6408; correct isotope distribution. Single crystalline specimen was obtained by slow diffusion of methanol into a chloroform solution of **3a**.

5,10,15,20-Tetrakis((tri*iso*propylsilyl)ethynyl)benzo[*i*]benzo[7,8]phenazino[2,1-*a*]phenazine (**3b**)

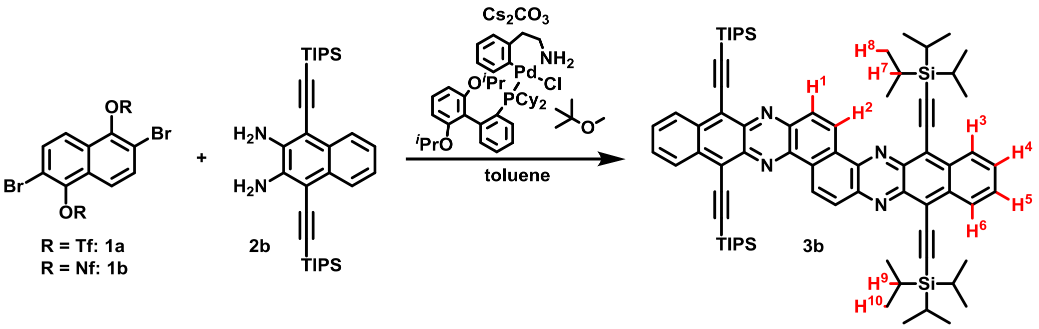

The **GP** was applied to **1a** (150 mg, 258 µmol, 1.00 equiv.) and **2b** (281 mg, 541 µmol, 2.10 equiv.) in 1 mL anhydrous, degassed toluene. Flash column chromatography (SiO_2; petroleum ether/diethyl ether 250:1 v/v) and gel permeation chromatography (toluene) yielded a green solid **3b** (140 mg, 121 µmol, 48%).

Applying the **GP** to **1b** (50.0 mg, 56.7 µmol, 1.00 equiv.) and **2b** (58.8 mg, 113 µmol, 2.00 equiv.) in 1 mL anhydrous, degassed toluene, flash column chromatography (SiO_2; petroleum ether/diethyl ether 250:1 v/v) and gel permeation chromatography (toluene) yielded a green solid **3b** (33.4 mg, 28.9 µmol, 51%).

R_f = 0.60 (SiO_2; petroleum ether/dichloromethane 2:1, v/v). Mp: ≥400°C (decomposition). ^{1}H-NMR (CDCl$_3$, 600.24 MHz, RT): δ = 9.95 (d, J = 9.32 Hz, 2H, H^2), 8.81 - 8.87 (m, 4H, H^3/H^6), 8.38 (d, J = 9.32 Hz, 2H, H^1), 7.71 - 7.75 (m, 4h, H^4/H^5), 1.40 - 1.43 (m, 42H, H H^7/H^8), 1.36 - 1.40 (m, 42H, H^9/H^{10}) ppm. ^{13}C-NMR (CDCl$_3$, 150.93 MHz, RT): δ = 145.4, 142.1, 141.5, 140.4, 135.6, 135.5, 132.6, 129.4, 129.4, 128.4, 128.4, 127.9, 127.8, 121.3, 121.1, 108.6, 108.1, 103.0, 102.9, 11.2, 19.1, 11.9, 11.8 ppm. IR (ATR): $\tilde{\nu}$ = 2941, 2862, 1461, 1386, 1239, 1044, 994, 881, 871, 757, 674, 663, 622, 595, 482, 457 cm^{-1}. HRMS (MALDI$^+$) m/z: [M+2H]$^+$: calcd. for [$C_{74}H_{98}N_4Si_4$]$^+$: 1154.6863; found 1154.6901; correct isotope distribution. Single crystalline specimen was obtained by slow diffusion of methanol into a chloroform solution of **3b**.

6,11,18,23-Tetrakis((tri*iso*propylsilyl)ethynyl)naphtho[2,3-*i*]naphtho[2',3':7,8]phenazino[2,1-*a*]phenazine (**3c**)

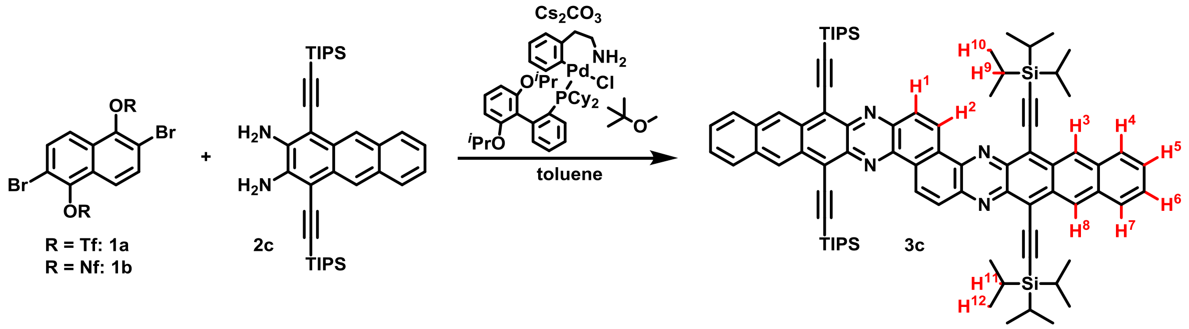

The **GP** was applied to **1a** (60.0 mg, 103 µmol, 1.00 equiv.) and **2c** (117 mg, 206 µmol, 2.00 equiv.) in 1 mL anhydrous, degassed toluene. Flash column chromatography (SiO_2; petroleum ether/diethyl ether 250:1 v/v) and gel permeation chromatography (toluene) yielded a brown solid **3c** (10.7 mg, 8.53 µmol, 8%).

Applying the **GP** to **1b** (100 mg, 113 µmol, 1.00 equiv.) and **2c** (129 mg, 227 µmol, 2.00 equiv.) in 1 mL anhydrous, degassed toluene, flash column chromatography (SiO_2; petroleum ether/diethyl ether 250:1 v/v) and gel permeation chromatography (toluene) yielded a brown solid **3c** (13.1 mg, 10.5 µmol, 9%).

R_f = 0.60 (SiO_2; petroleum ether/dichloromethane 2:1, v/v). Mp: ≥400°C (decomposition). ^{1}H-NMR (CDCl$_3$, 600.24 MHz, RT): δ = 9.89 (d, *J* = 9.28 Hz, 2H, H^2), 9.54 (s, 2H, H^3/H^8), 9.50 (s, 2H, H^3/H^8), 8.23 (d, *J* = 9.28 Hz, 2H, H^1), 8.06-8.10 (m, 4H, H^4/H^7), 7.52 - 7.55 (m, 4H, H^5/H^6), 1.46 - 1.49 (m, 42H, H^9/H^{10}), 1.41 - 1.44 (s, 42H, H^{11}/H^{12}) ppm. IR (ATR): $\tilde{\nu}$ = 2940, 2862, 1462, 1374, 996, 880, 848, 740, 673, 585 cm^{-1}. HRMS (MALDI$^+$) m/z: [M+2H]$^+$: calcd. for $[C_{82}H_{102}N_4Si_4]^+$: 1254.7176; found 1254.7215; correct isotope distribution.

S3. Catalyst screening

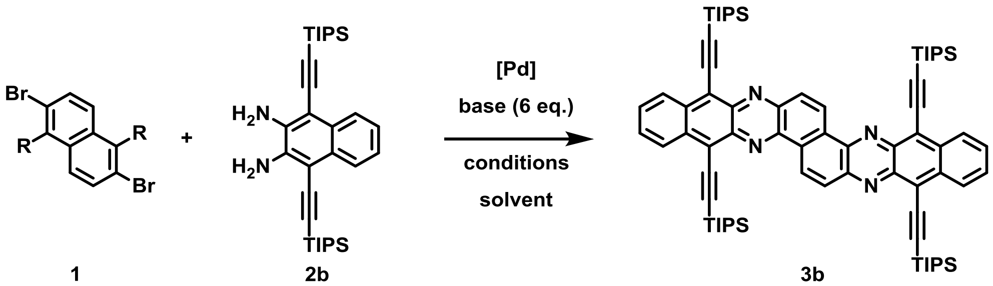

1 2b 3b

Table S1. Screening of reaction conditions for the Buchwald-Hartwig coupling reaction of **1** with **2b** yielding **3b**.

Compd	Catalyst	Base	Solvent	Temperature	Reaction Time	Yield
**1a**	Pd(dba)$_2$/ dppf	NaO^{t}Bu	toluene	85 °C	24 h	decomposition
**1a**	Pd(dba)$_2$/ BINAP	K_3PO_4	toluene	85 °C	24 h	decomposition
**1a**	Pd(dba)$_2$/ dppf	Cs_2CO_3	toluene	85 °C	24 h	5%
**1a**	RuPhos G1	Cs_2CO_3	toluene	85 °C	24 h	30%
**1a**	RuPhos G1	Cs_2CO_3	1,4-dioxane	120 °C/ microwave	16 h	33%
**1a**	RuPhos G1	Cs_2CO_3	toluene	120 °C/ microwave	16 h	37%
**1a**	RuPhos G1	Cs_2CO_3	toluene	110 °C	24 h	48%
**1a**	RuPhos G1	Cs_2CO_3	toluene	110 °C	72 h	23%
**1b**	RuPhos G1	Cs_2CO_3	toluene	110 °C	24 h	51%

S4. UV-Vis spectra

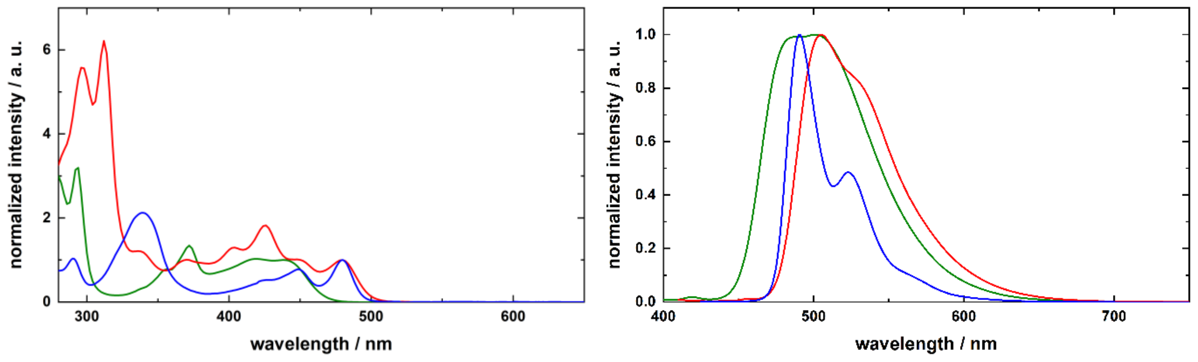

Figure S1: Absorption (left) and emission (right) spectrum of **4a** (green), **3a** (red), **5a** (blue) in DCM.

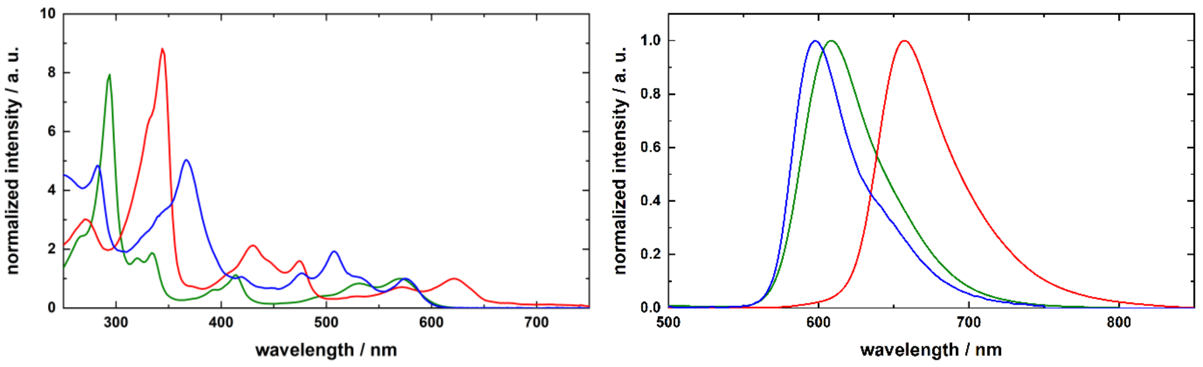

Figure S2: Absorption (left) and emission (right) spectrum of **4b** (green), **3b** (red), **5b** (blue) in DCM.

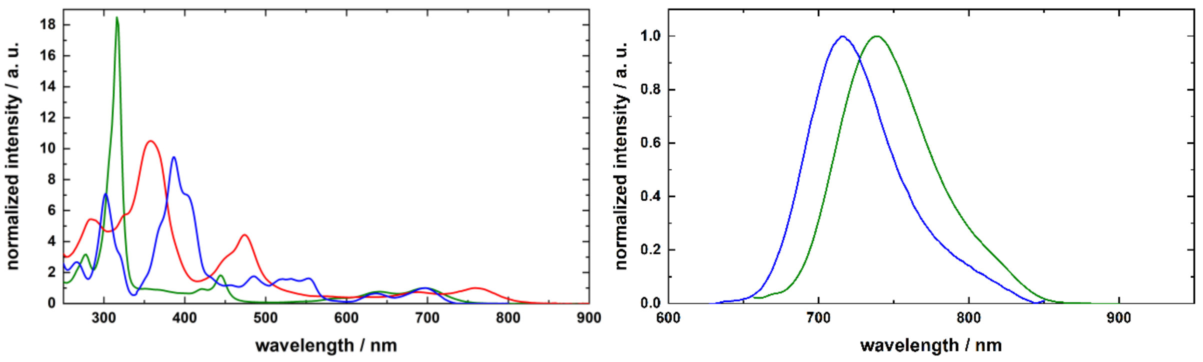

Figure S3: Absorption (left) and emission (right) spectrum of **4c** (green), **3c** (red), **5c** (blue) in DCM. **3c** is nonfluorescent.

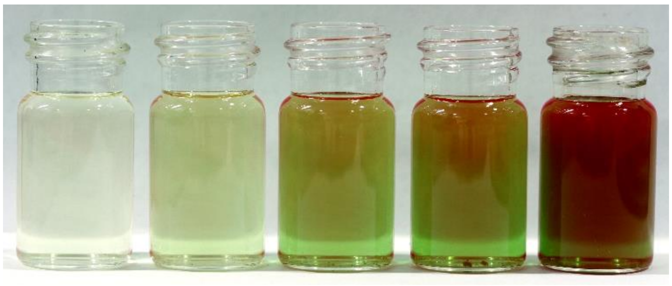

Figure S4: Photographs of **3b** in different concentrations in hexane. Already at relatively low concentrations the strong fluorescence of **3b** starts to overwhelm the true color (green) of the solution even in daylight.

S5. UV-Vis absorption stability study

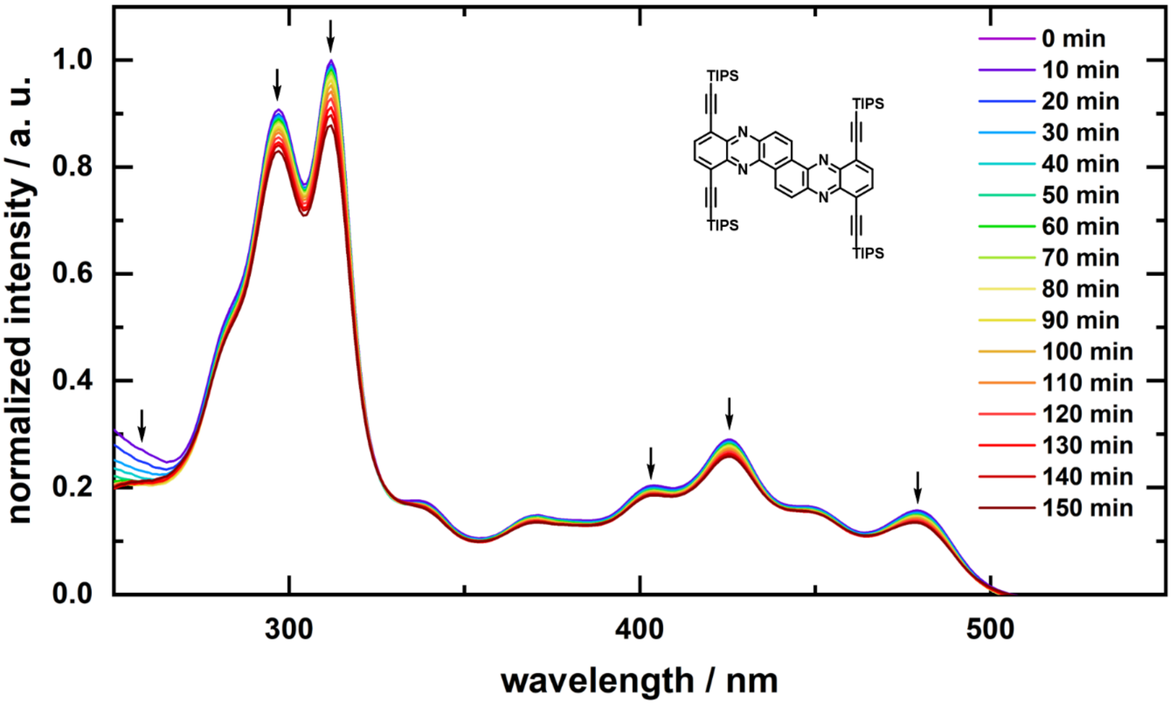

Figure ***S***5***:*** Change in absorption intensity of a solution of 3a (10^{-6}M) after irradiation with a handheld UV lamp (λ = 365 nm) in DCM at room temperature under ambient conditions.

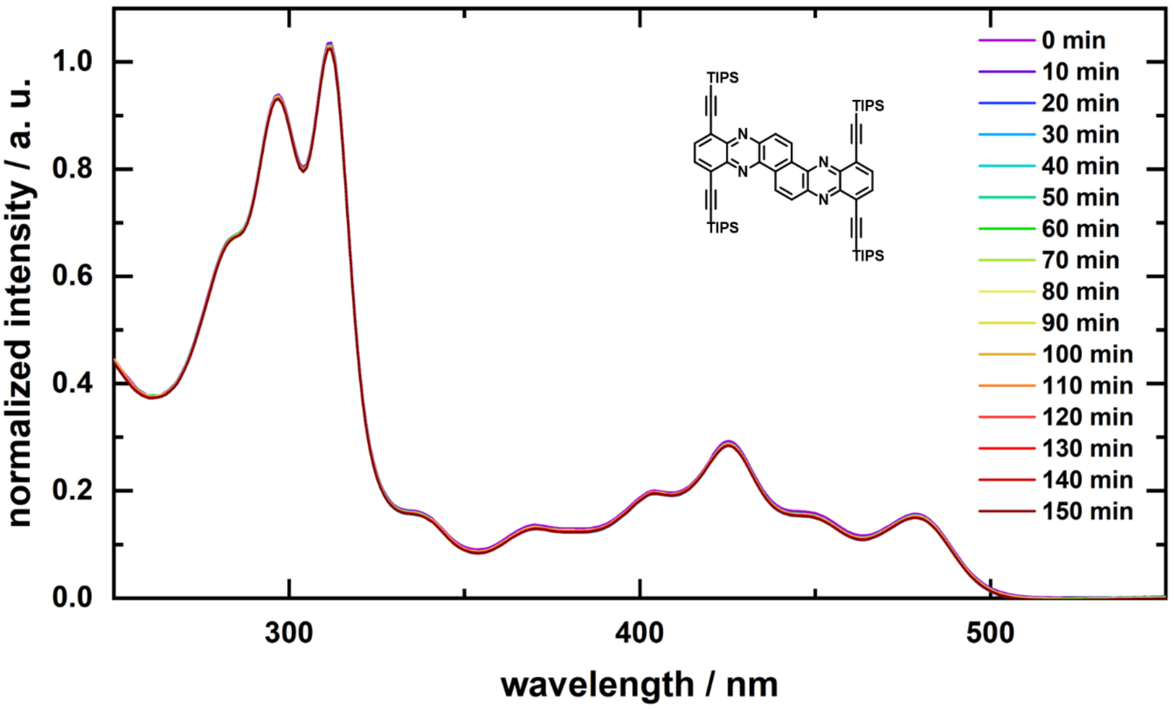

Figure ***S6:*** Change in absorption intensity of a solution of **3a** (10^{-6}M) after irradiation with a handheld UV lamp (λ = 365 nm) in DCM at room temperature under nitrogen atmosphere.

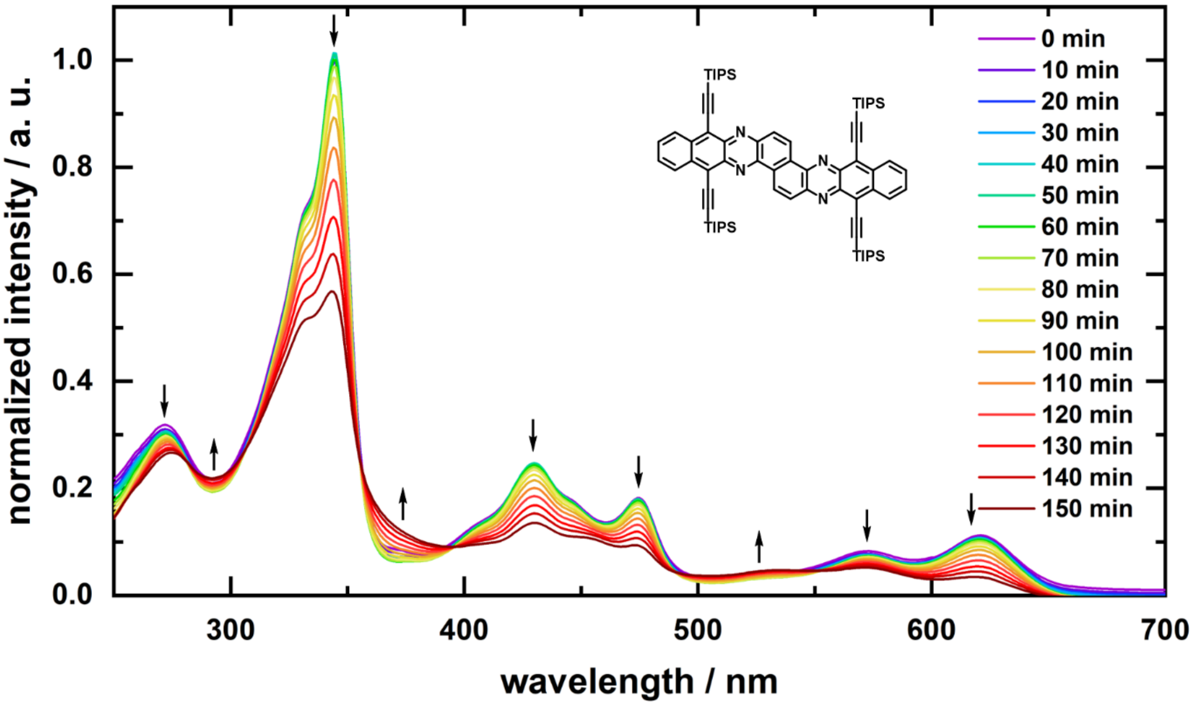

Figure 7*:* Change in absorption intensity of a solution of **3b** (10^{-6}M) after irradiation with a handheld UV lamp (λ = 365 nm) in DCM at room temperature under ambient conditions.

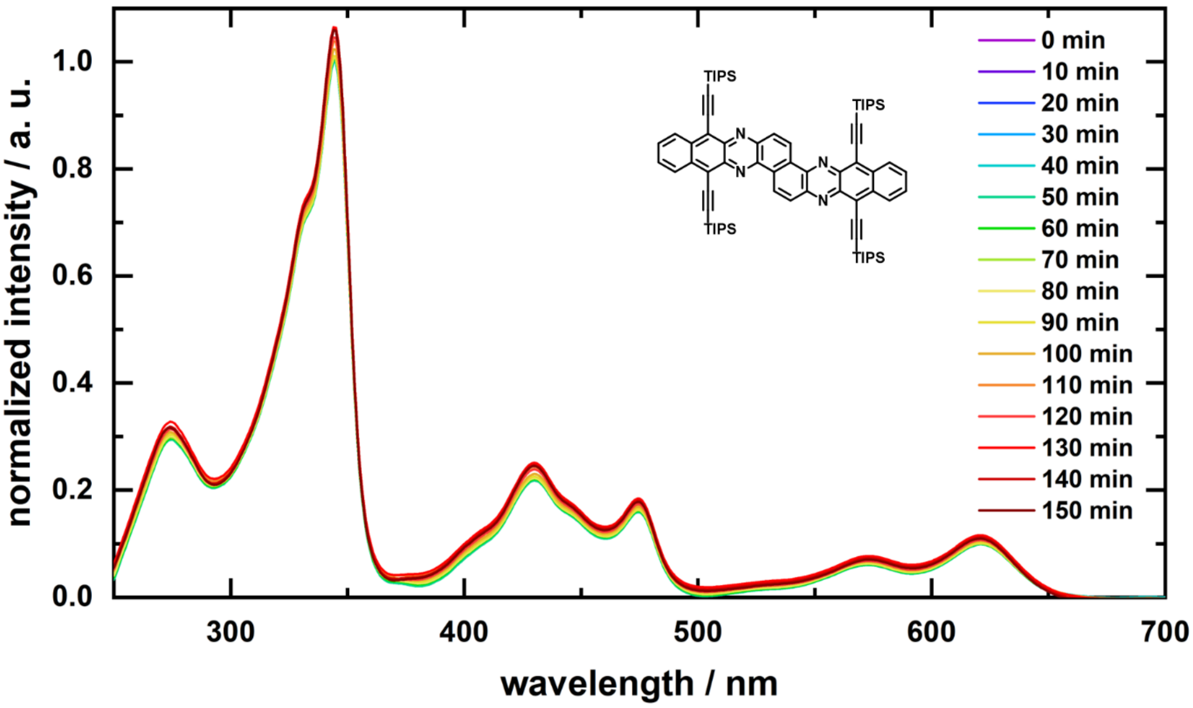

Figure 8*:* Change in absorption intensity of a solution of **3b** (10^{-6}M) after irradiation with a handheld UV lamp (*λ* = 365 nm) in DCM at room temperature under nitrogen atmosphere.

S6. Electrochemistry/cyclic voltammetry

The cyclic voltammetry (CV) experiments were carried out using a platinum working electrode, a platinum/titanium wire auxiliary electrode, a silver wire reference electrode, a 0.1 $molL^{-1}$ NBu_4PF_6 solution in degassed dry THF, and ferrocene/ferrocenium as the reference redox system and internal standard (−4.8 eV) at room temperature and 0.2 mVs^{-1}. To determine the first reduction potentials ($E_{(0/–)}$) of **3a-c** and the first oxidation potential of ferrocene, the half-wave potentials were used.[7]

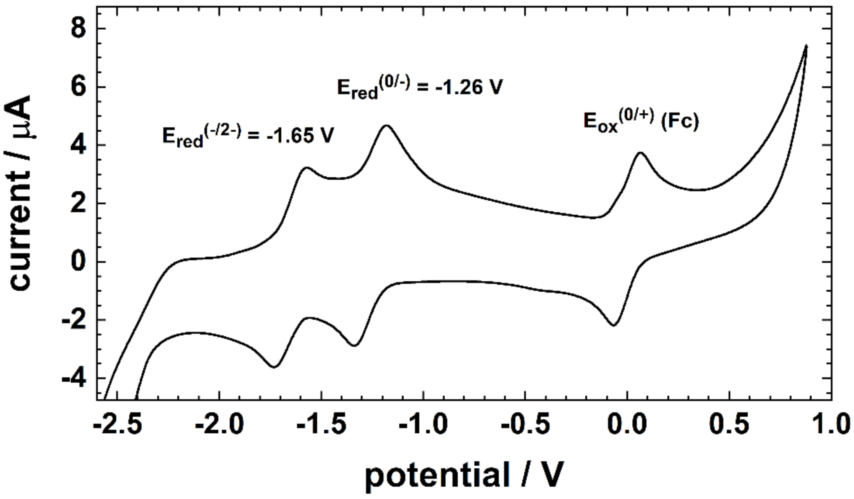

Figure S9: CV spectrum of 1,4,9,12-tetrakis((tri*iso*propylsilyl)ethynyl)phenazino[2,1-*a*] phenazine (**3a**).

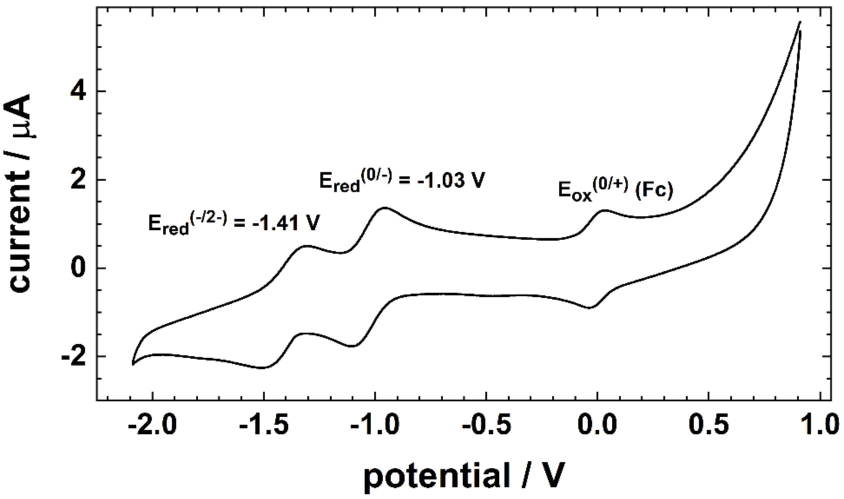

Figure S10: CV spectrum of 5,10,15,20-tetrakis((tri*iso*propylsilyl)ethynyl)benzo[*i*]benzo[7,8] phenazino-[2,1-*a*]phenazine (**3b**).

S7. Quantum-chemical calculations of the FMOs

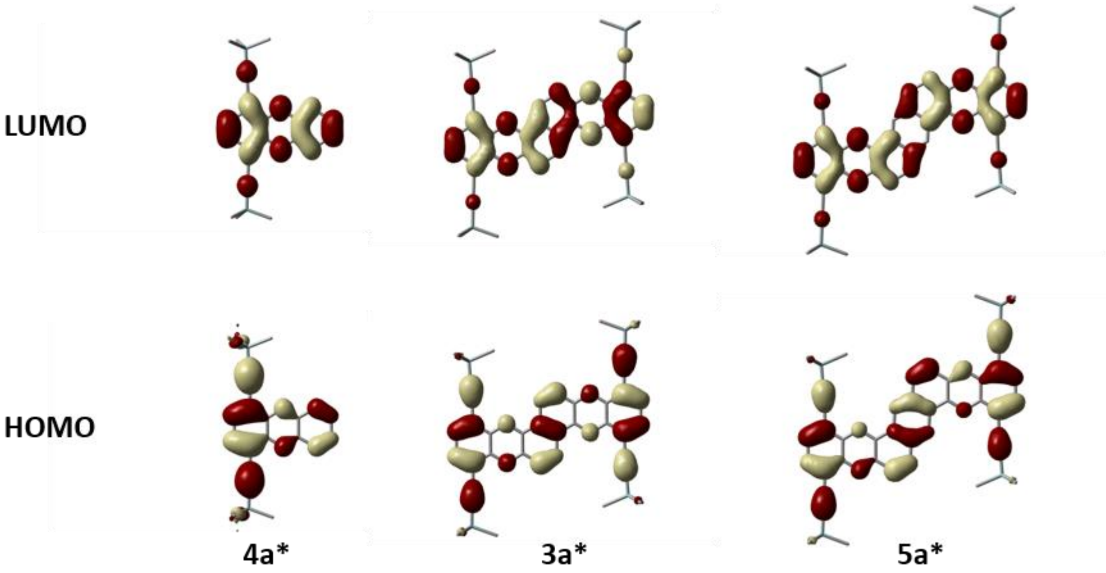

Figure S11: Quantum chemical calculations (TURBOMOLE B3LYP/def2-TZVP//Gaussian09 B3LYP/6-311++G**) of the FMOs (LUMOs top, HOMOs bottom) for compounds **3a**, **4a**, and **5a**. TMS groups were used instead of TIPS.

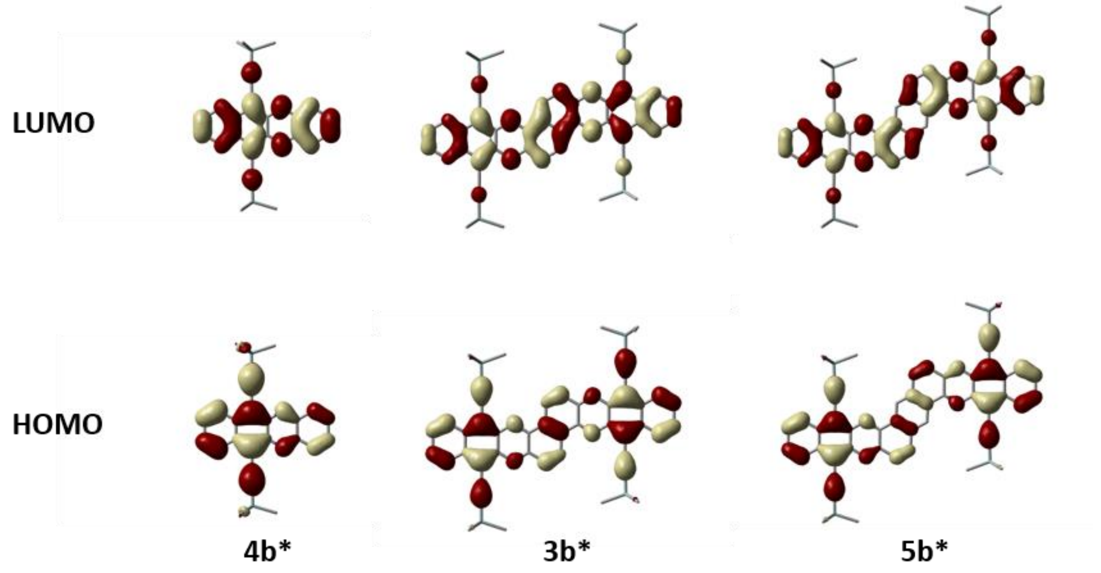

Figure S12: Quantum chemical calculations (TURBOMOLE B3LYP/def2-TZVP//Gaussian09 B3LYP/6-311++G**) of the FMOs (LUMOs top, HOMOs bottom) for compounds **3b**, **4b**, and **5b**. TMS groups were used instead of TIPS.

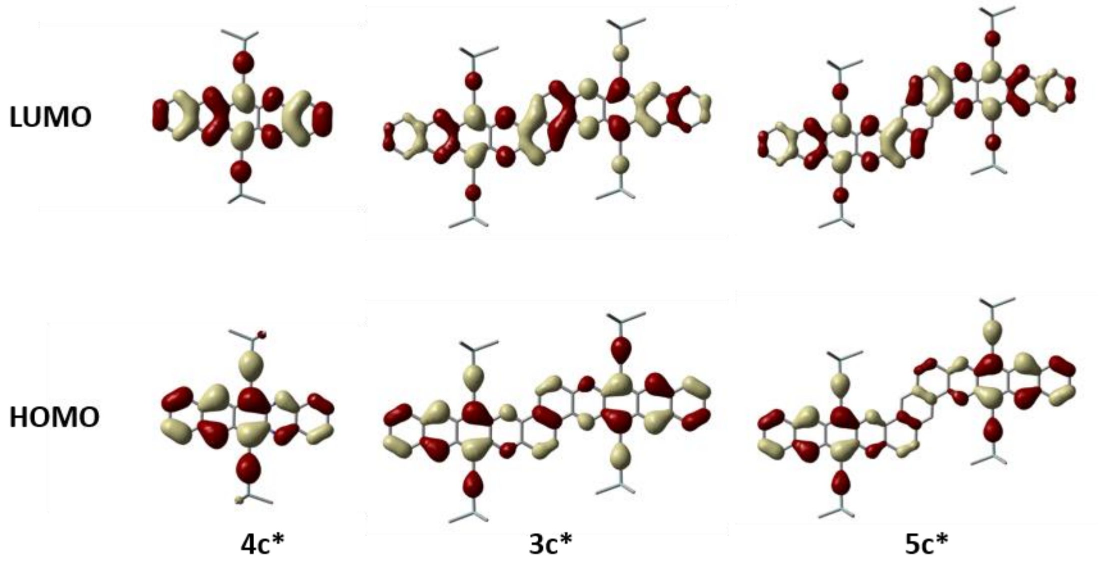

Figure S13: Quantum chemical calculations (TURBOMOLE B3LYP/def2-TZVP//Gaussian09 B3LYP/6-311++G**) of the FMOs (LUMOs top, HOMOs bottom) for compounds **3c**, **4c**, and **5c**. TMS groups were used instead of TIPS.

S8. NMR spectra

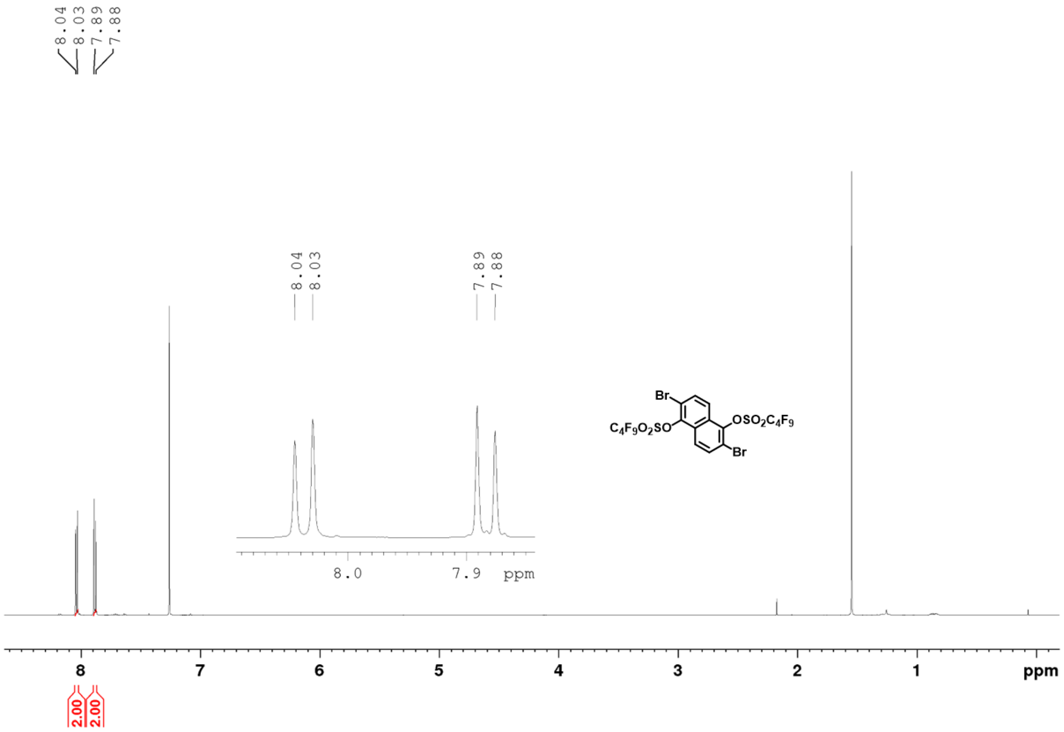

Figure S14: ^{1}H-NMR spectrum (600 MHz) of **1b** in $CDCl_3$.

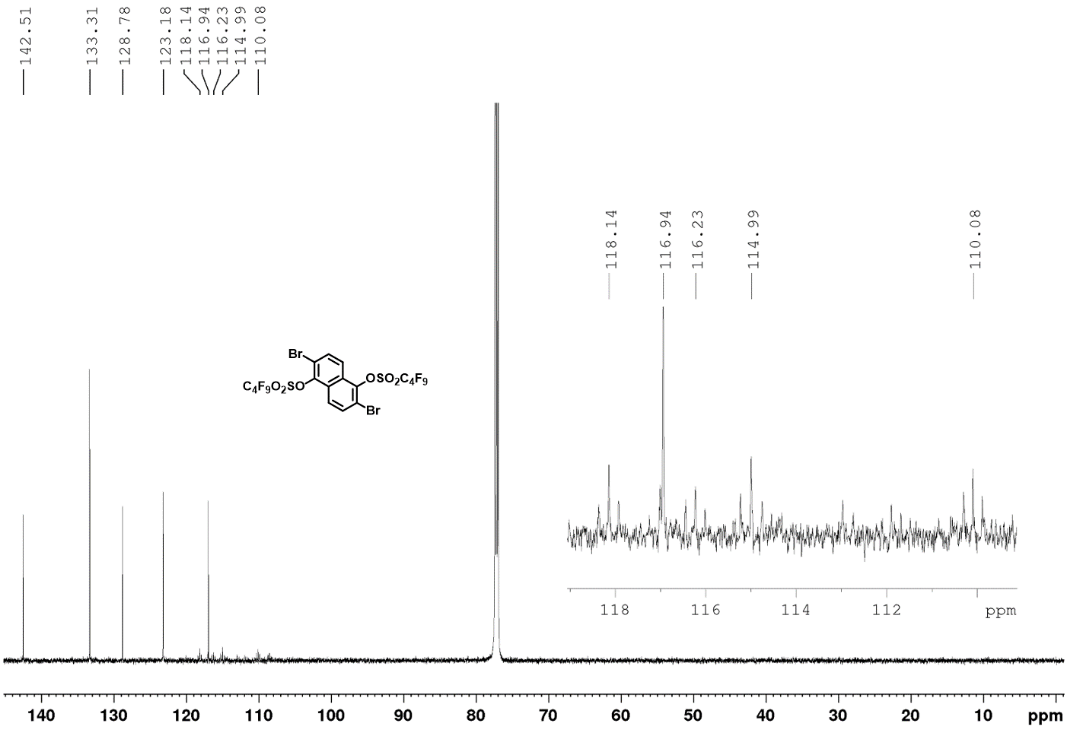

Figure S15: $^{13}C\{^{1}H\}$-NMR spectrum (151 MHz) of **1b** in $CDCl_3$.

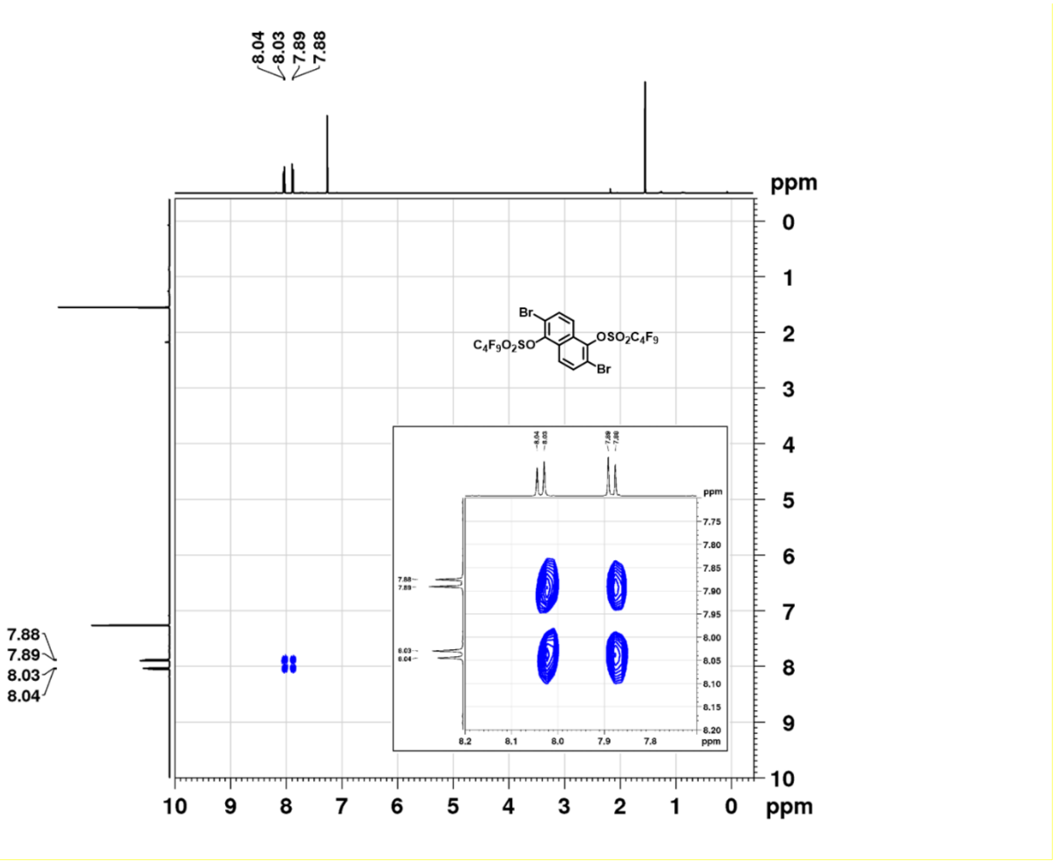

Figure S16: $^{1}H,^{1}H$-COSY-NMR spectrum (500 MHz) of **1b** in $CDCl_3$.

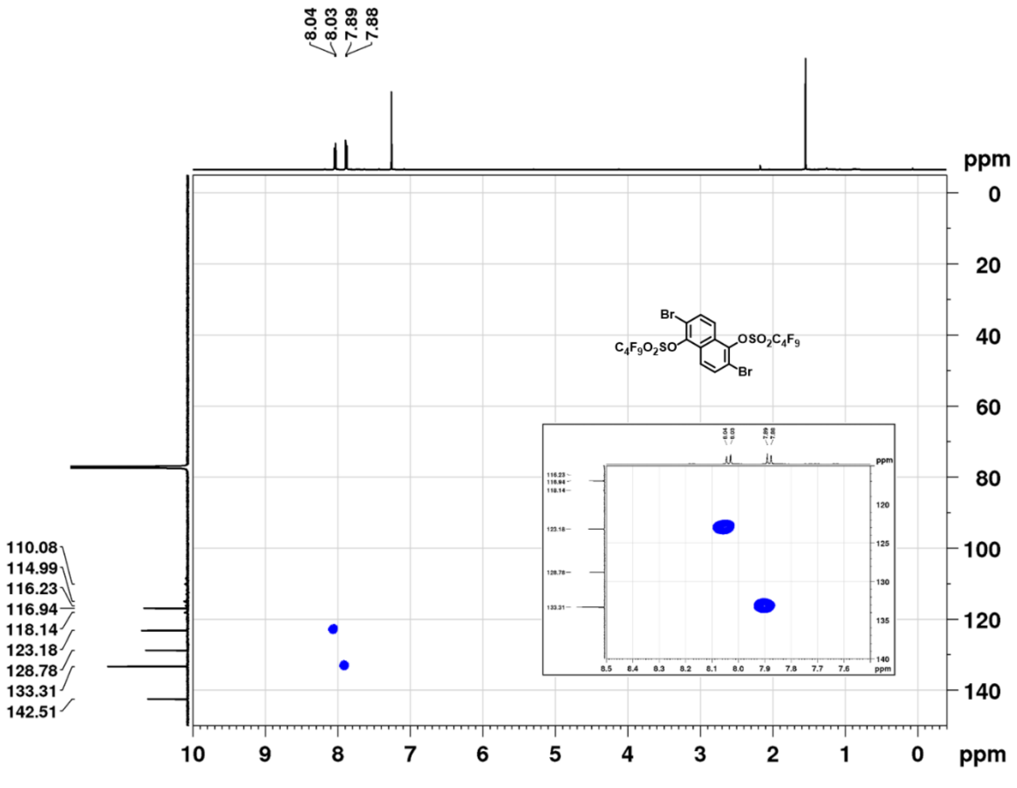

Figure S17: $^{1}H,^{13}C$-HSQC-NMR spectrum (500 MHz/126 MHz) of **1b** in $CDCl_3$.

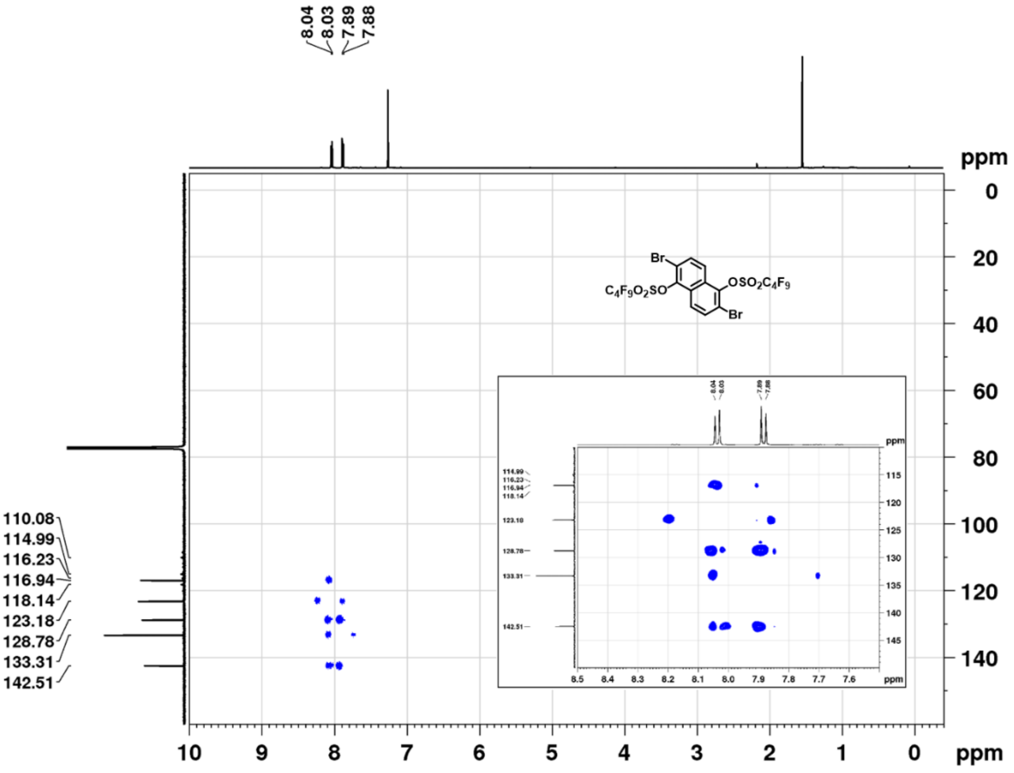

Figure S18: $^{1}H,^{13}C$-HMBC-NMR spectrum (500 MHz/126 MHz) of **1b** in $CDCl_3$.

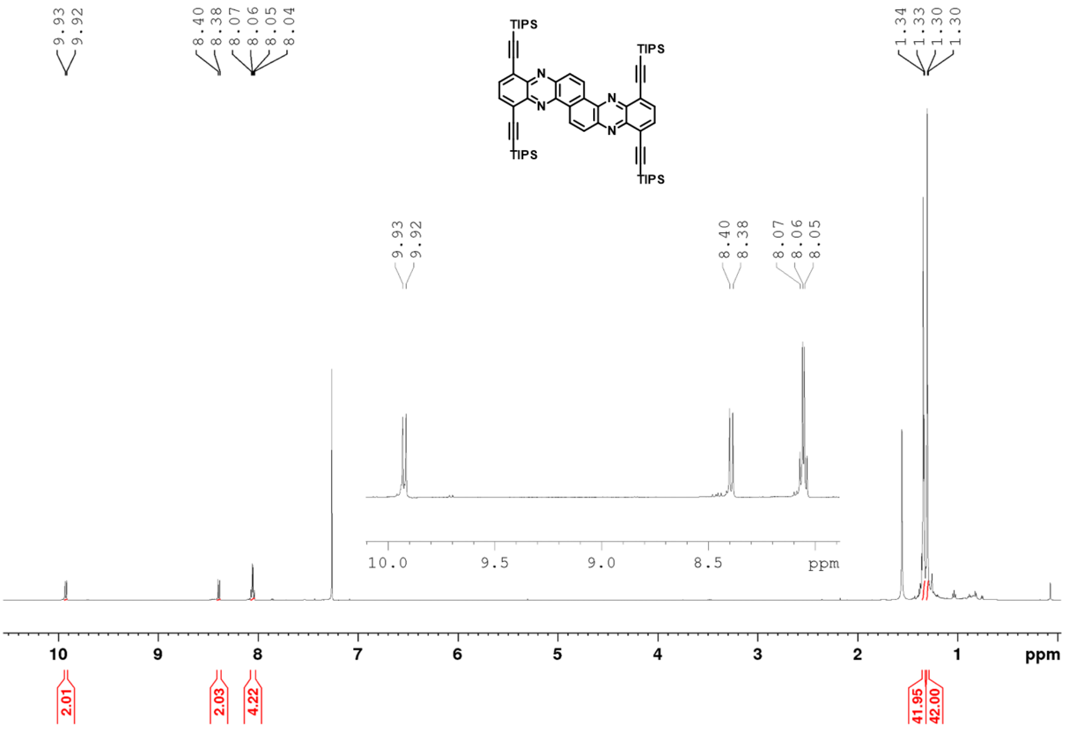

Figure S19: ^{1}H-NMR spectrum (600 MHz) of **3a** in $CDCl_3$.

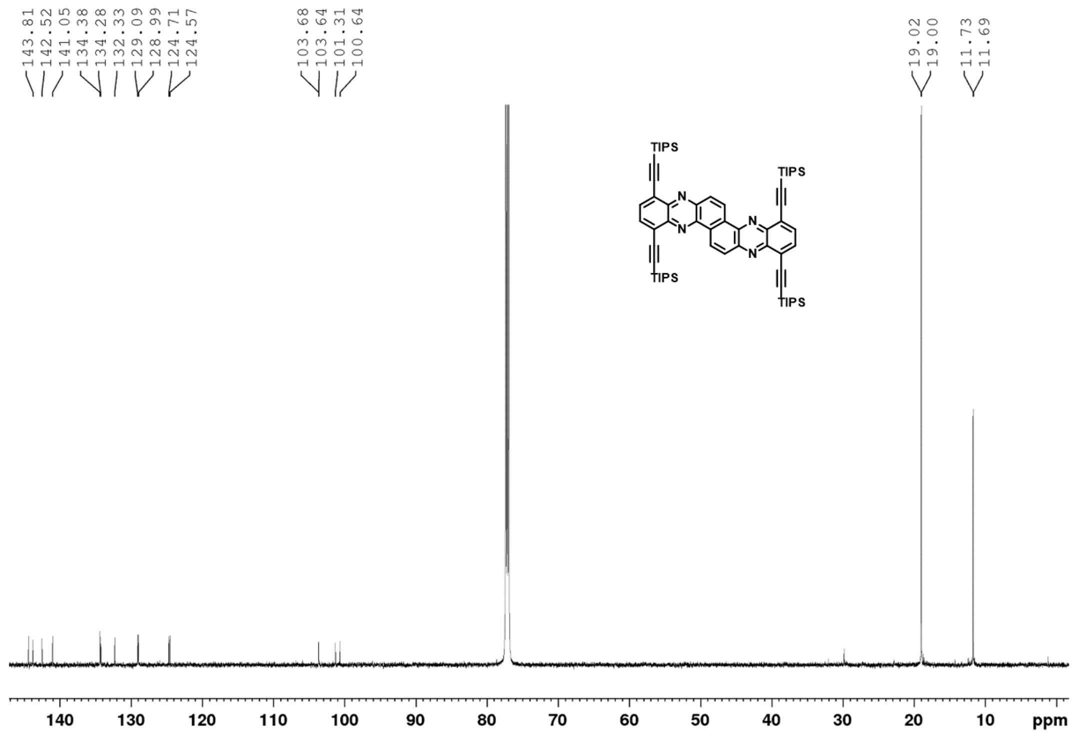

Figure S20: $^{13}C\{^{1}H\}$-NMR spectrum (151 MHz) of **3a** in $CDCl_3$.

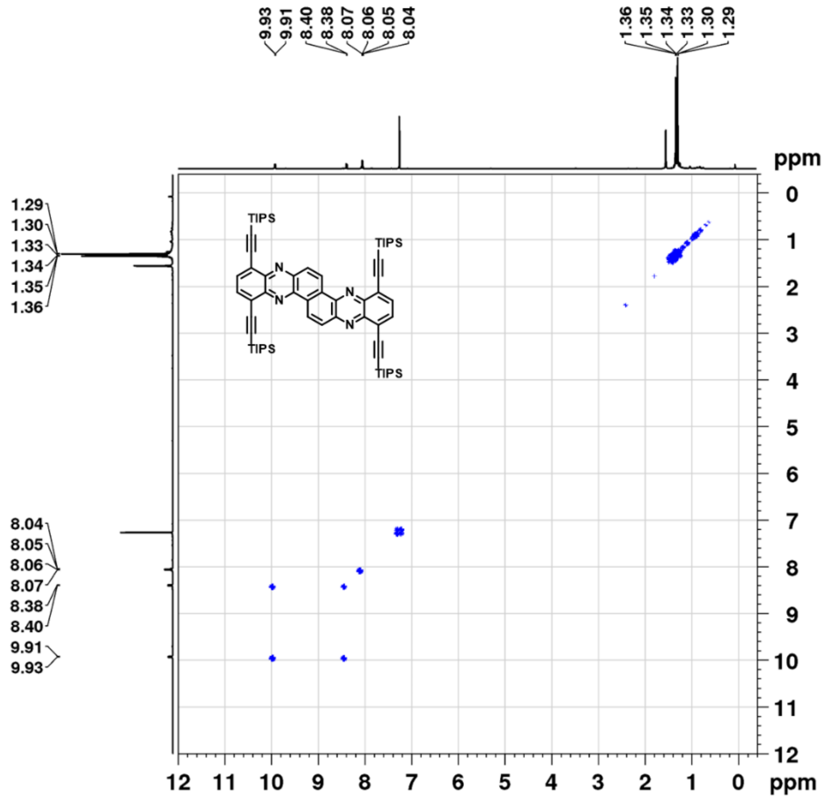

Figure S21: ^{1}H,^{1}H-COSY-NMR spectrum (600 MHz) of **3a** in $CDCl_3$.

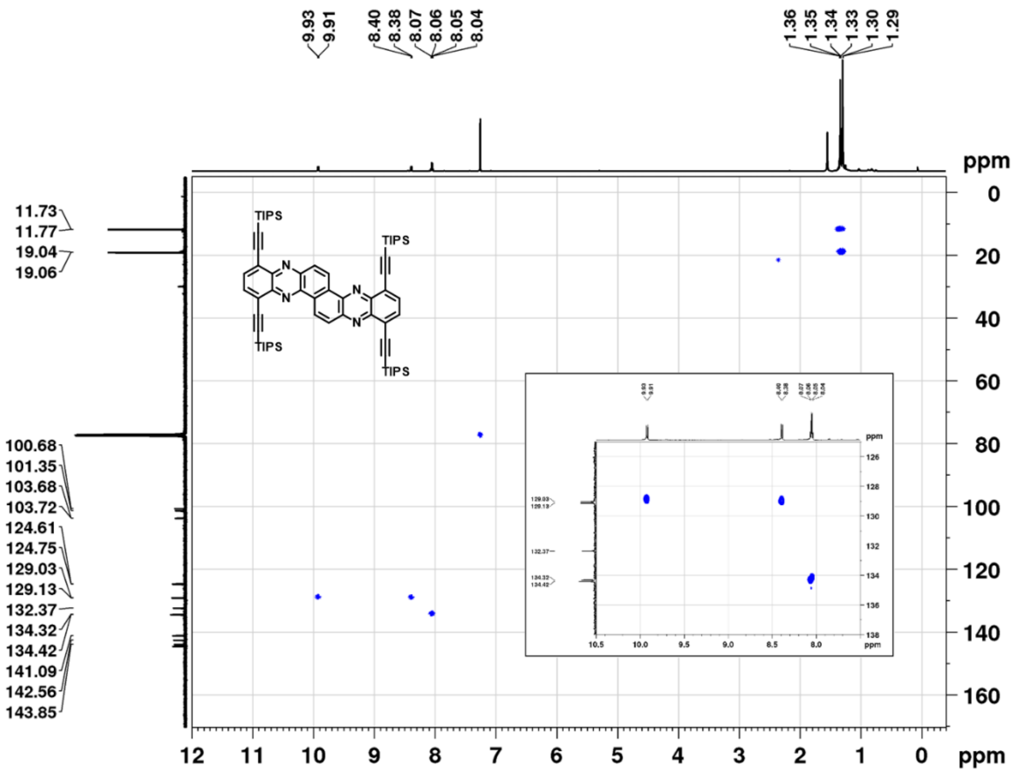

Figure S22: $^{1}H,^{13}C$-HSQC-NMR spectrum (600 MHz/151 MHz) of **3a** in $CDCl_3$.

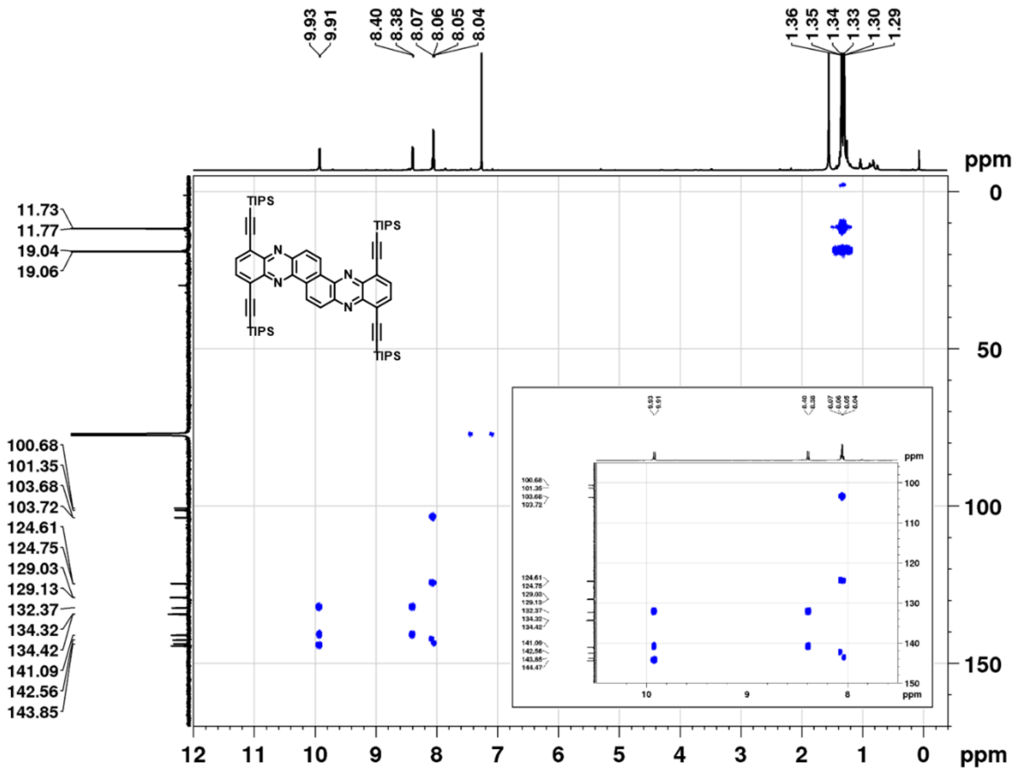

Figure S23: $^{1}H,^{13}C$-HMBC-NMR spectrum (600 MHz/151 MHz) of **3a** in $CDCl_3$.

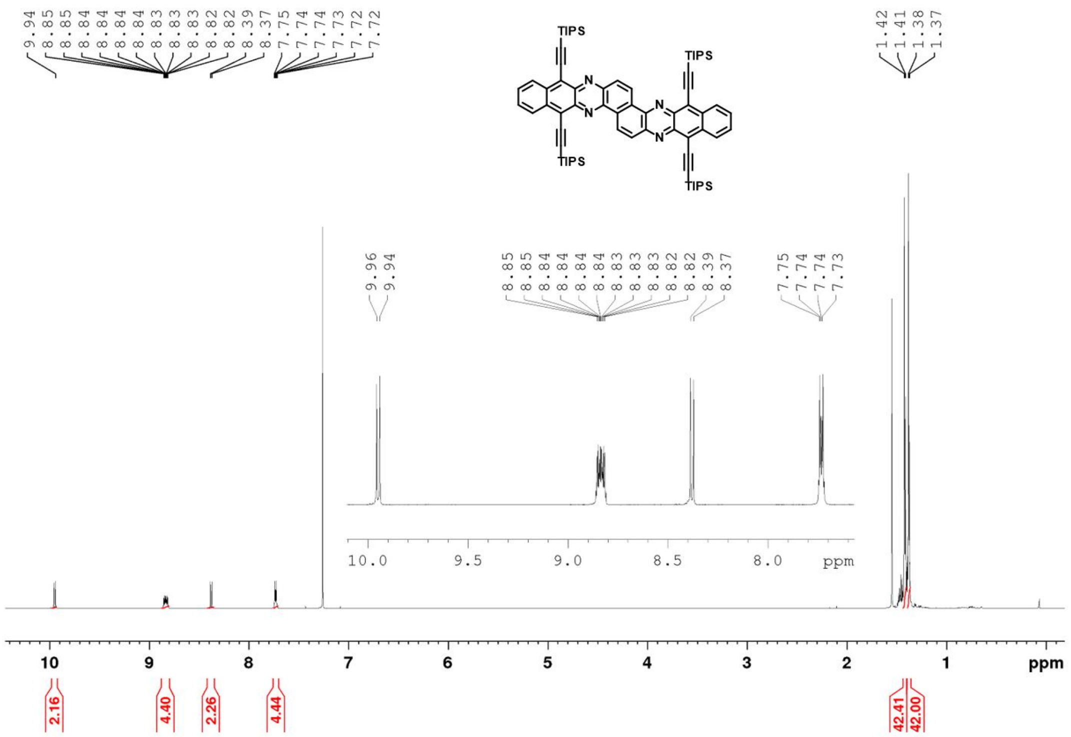

Figure S24: ^{1}H-NMR spectrum (600 MHz) of **3b** in $CDCl_3$.

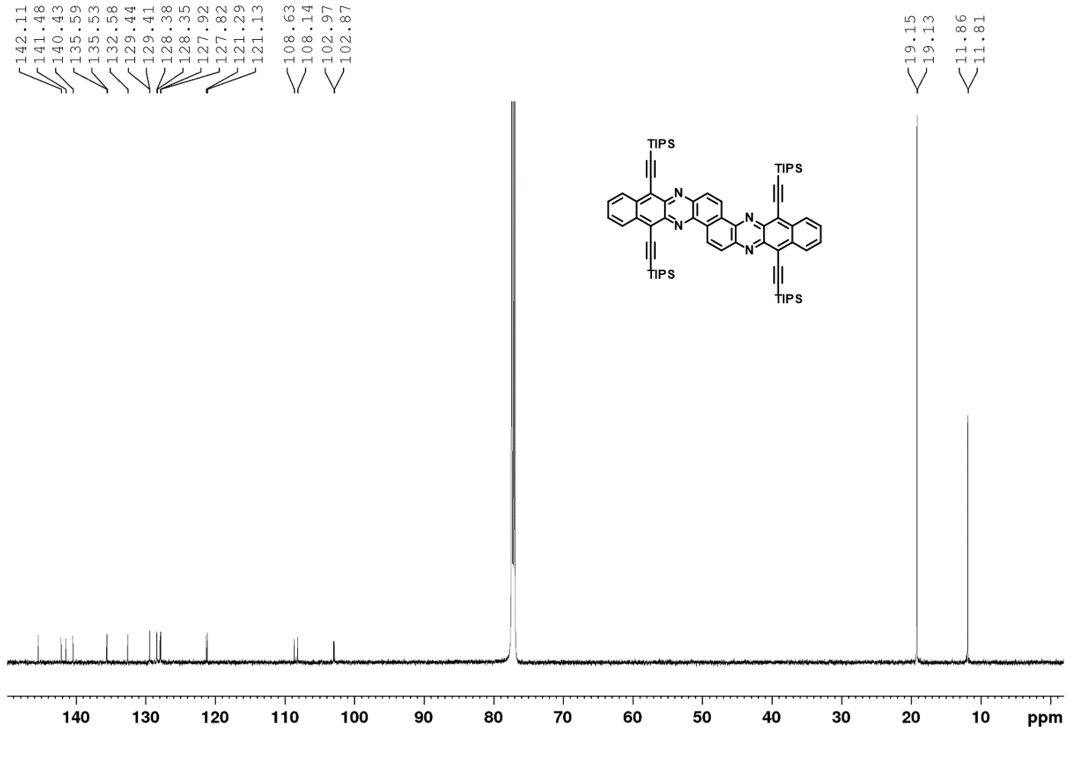

Figure S25: $^{13}C\{^1H\}$-NMR spectrum (151 MHz) of **3b** in $CDCl_3$.

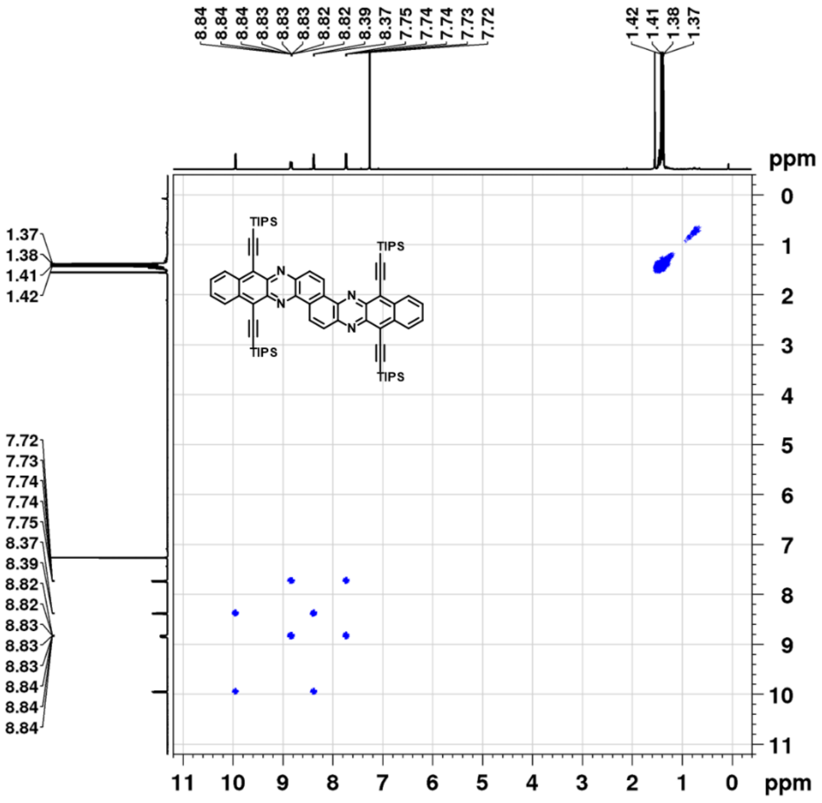

Figure S26: ^{1}H,^{1}H-COSY-NMR spectrum (600 MHz) of **3b** in $CDCl_3$.

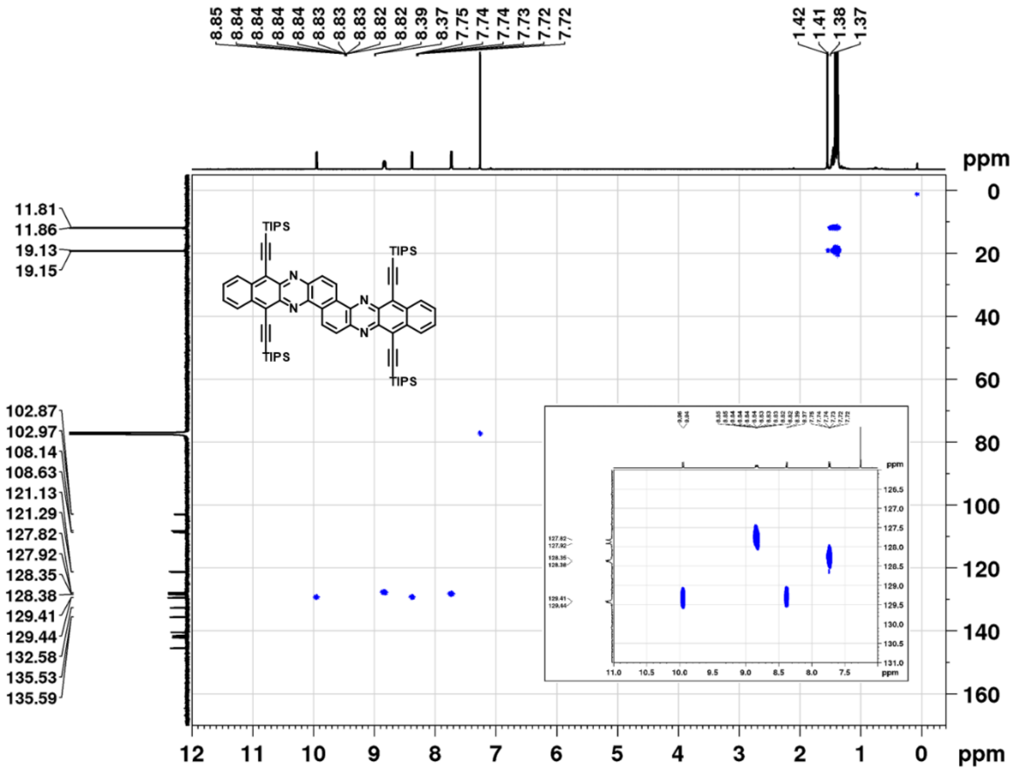

Figure S27: ^{1}H,^{13}C-HSQC-NMR spectrum (600 MHz/151 MHz) of **3b** in $CDCl_3$.

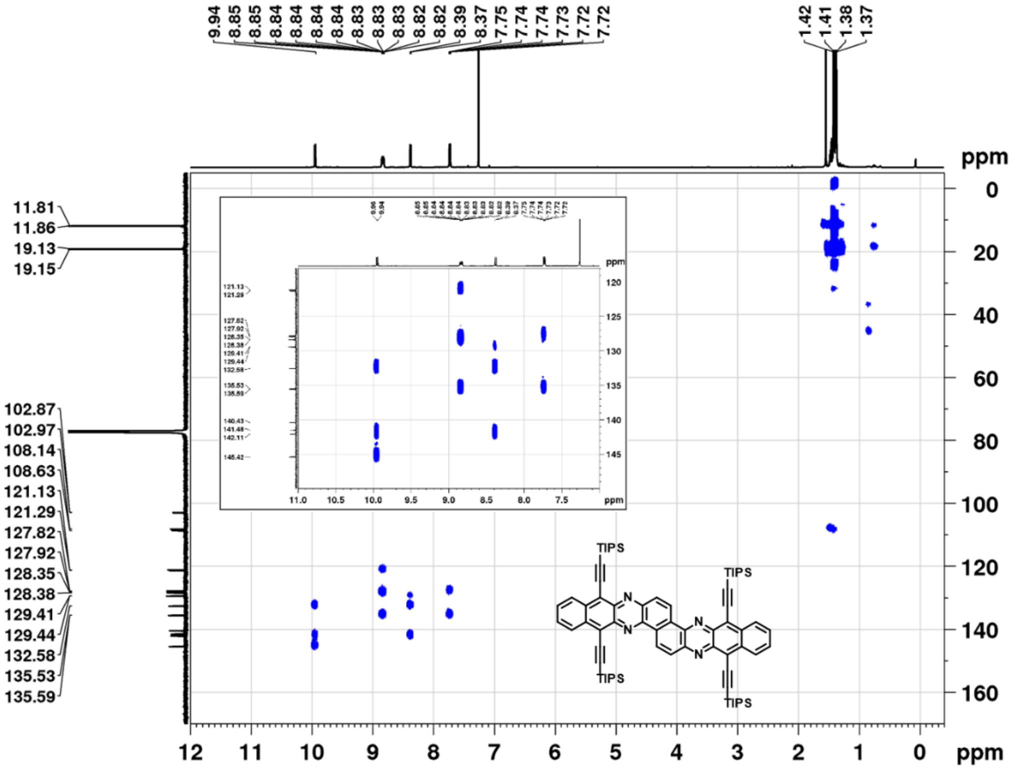

Figure S28: ^{1}H,^{13}C-HMBC-NMR spectrum (600 MHz/151 MHz) of **3b** in $CDCl_3$.

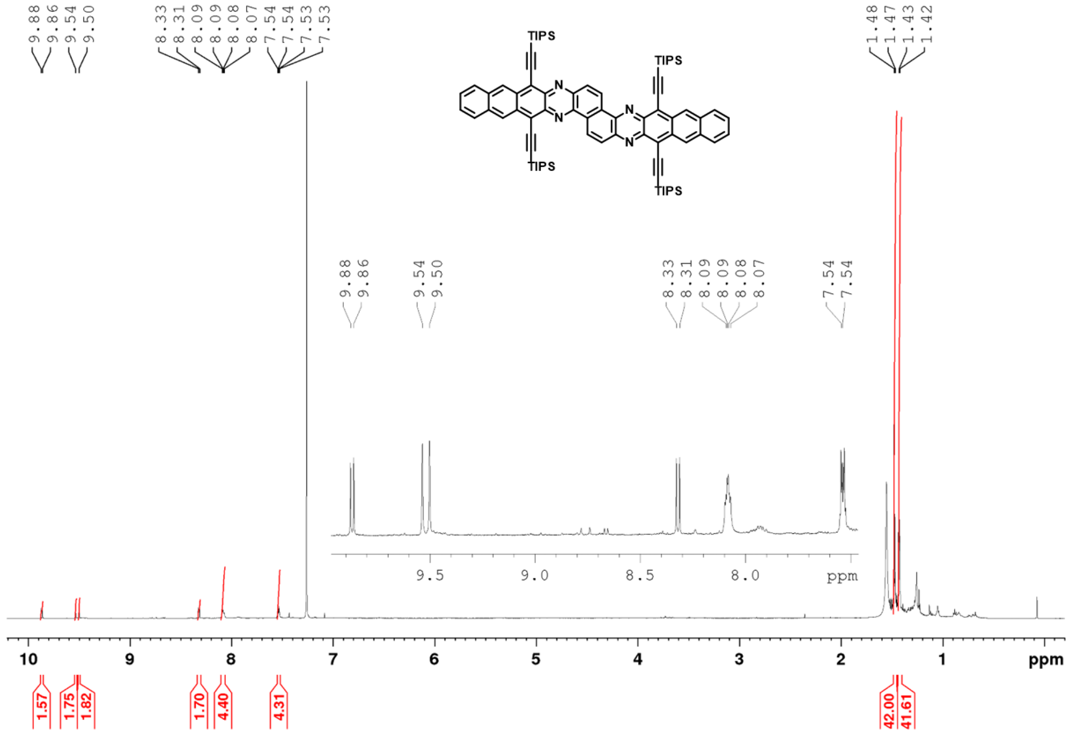

Figure S29: ^{1}H-NMR spectrum (600 MHz) of 3c in $CDCl_3$.

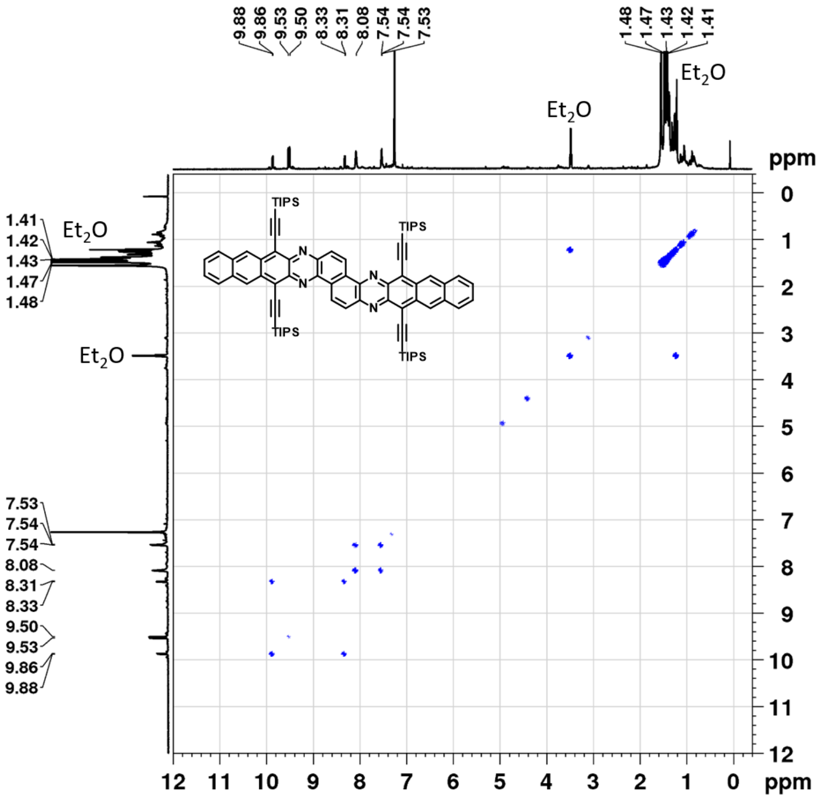

Figure S30: $^{1}H,^{1}H$-COSY-NMR spectrum (600 MHz) of **3c** in $CDCl_3$.

S9. X-ray single-crystal structure analysis

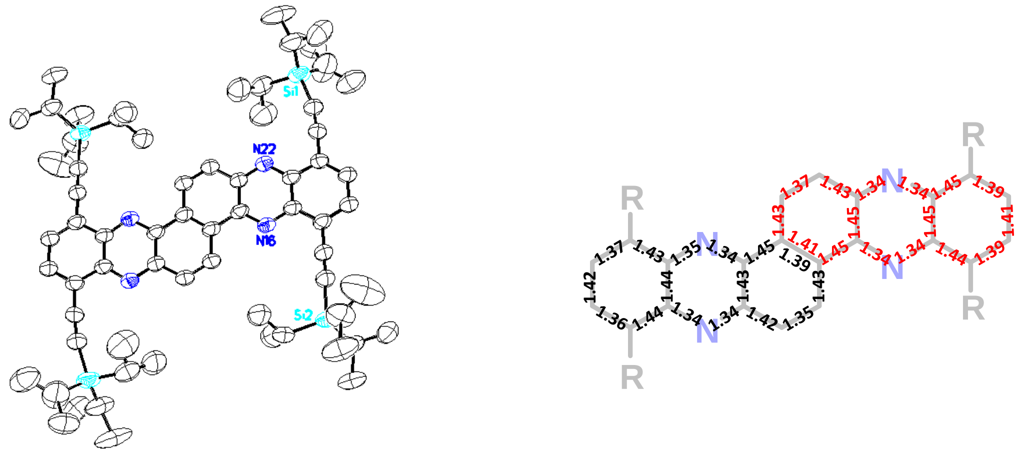

Figure S31*:* Left: ORTEP structure of **3a** (CCDC 1946373); right: bond lengths, measured in black, calculated (TURBOMOLE B3LYP/def2-TZVP) in red.

Empirical formula	$C_{66}H_{92}N_4Si_4$
Formula weight	1053.79
Temperature	200(2) K
Wavelength	1.54178 Å
Crystal system	triclinic
Space group	$P\bar{1}$
Z	1
Unit cell dimensions	a = 10.7023(6) Å α = 108.434(5) deg.
	b = 12.3000(8) Å β = 91.691(4) deg.
	c = 12.8404(7) Å γ = 98.192(5) deg.
Volume	1582.18(17) Å^3
Density (calculated)	1.11 g/cm^3
Absorption coefficient	1.17 mm^{-1}
Crystal shape	plank
Crystal size	0.203 x 0.044 x 0.019 mm^3
Crystal colour	brown

Theta range for data collection	3.8 to 62.2 deg.
Index ranges	-12≤h≤10, -13≤k≤14, -14≤l≤11
Reflections collected	12488
Independent reflections	4792 (R(int) = 0.0440)
Observed reflections	2909 (I > 2σ(I))
Absorption correction	Semi-empirical from equivalents
Max. and min. transmission	1.42 and 0.63
Refinement method	Full-matrix least-squares on F^2
Data/restraints/parameters	4792 / 1989 / 483
Goodness-of-fit on F^2	1.07
Final R indices (I>2sigma(I))	R1 = 0.089, wR2 = 0.168
Largest diff. peak and hole	0.43 and -0.25 eÅ^{-3}

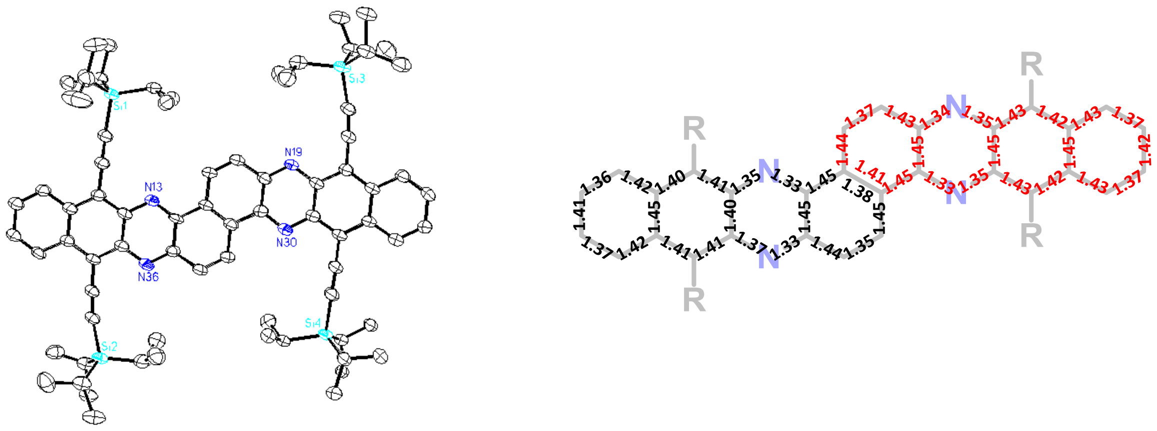

Figure S32*:* Left: ORTEP structure of **3b** (CCDC 1946372); right: bond lengths, measured in black, calculated (TURBOMOLE B3LYP/def2-TZVP) in red.

Empirical formula	$C_{74}H_{96}N_4Si_4$
Formula weight	1153.90
Temperature	110(2) K
Wavelength	1.54178 Å
Crystal system	triclinic

Space group	$P\bar{1}$
Z	2
Unit cell dimensions	a = 12.2249(8) Å α = 71.693(5) deg.
	b = 16.5676(10) Å β = 86.957(5) deg.
	c = 17.9511(11) Å γ = 87.140(5) deg.
Volume	3444.8(4) Å^3
Density (calculated)	1.11 g/cm^3
Absorption coefficient	1.12 mm^{-1}
Crystal shape	cubic
Crystal size	0.089 x 0.053 x 0.046 mm^3
Crystal colour	violet
Theta range for data collection	2.8 to 67.1 deg.
Index ranges	$-14\leq h\leq 13$, $-19\leq k\leq 13$, $-20\leq l\leq 21$
Reflections collected	28544
Independent reflections	11684 (R(int) = 0.0523)
Observed reflections	6610 (I > 2σ(I))
Absorption correction	Semi-empirical from equivalents
Max. and min. transmission	1.87 and 0.56
Refinement method	Full-matrix least-squares on F^2
Data/restraints/parameters	11684 / 1304 / 791
Goodness-of-fit on F^2	1.04
Final R indices (I>2sigma(I))	R1 = 0.085, wR2 = 0.177
Largest diff. peak and hole	0.49 and -0.49 eÅ^{-3}

[1] G. R. Fulmer, A. J. Miller, N. H. Sherden, H. E. Gottlieb, A. Nudelman, B. M. Stoltz, J. E. Bercaw, K. I. Goldberg, *Organometallics* **2010,** *29*, 2176-2179.

[2] Gaussian 09, Revision A.02, M. J. Frisch, G. W. Trucks, H. B. Schlegel, G. E. Scuseria, M. A. Robb, J. R. Cheeseman, G. Scalmani, V. Barone, B. Mennucci, G. A. Petersson, H. Nakatsuji, M. Caricato, X. Li, H. P. Hratchian, A. F. Izmaylov, J. Bloino, G. Zheng, J. L. Sonnenberg, M. Hada, M. Ehara, K. Toyota, R. Fukuda, J. Hasegawa, M. Ishida, T. Nakajima, Y. Honda, O. Kitao, H. Nakai, T. Vreven, J. A. Montgomery, Jr., J. E. Peralta, F. Ogliaro, M. Bearpark, J. J. Heyd, E. Brothers, K. N. Kudin, V. N. Staroverov, R. Kobayashi, J. Normand, K. Raghavachari, A. Rendell, J. C. Burant, S. S. Iyengar, J. Tomasi, M. Cossi, N. Rega, J. M. Millam, M. Klene, J. E. Knox, J. B. Cross, V. Bakken, C. Adamo, J. Jaramillo, R. Gomperts, R. E. Stratmann, O. Yazyev, A. J. Austin, R. Cammi, C. Pomelli, J. W. Ochterski, R. L. Martin, K. Morokuma, V. G. Zakrzewski, G. A. Voth, P. Salvador, J. J. Dannenberg, S. Dapprich, A. D. Daniels, Ö. Farkas, J. B. Foresman, J. V. Ortiz, J. Cioslowski, D. J. Fox, Gaussian, Inc. Wallingford CT, **2009**.

[3] Y. Lu, Y. Qiao, H. Xue, G. Zhou, *Org. Lett.* **2018**, *20*, 6632-6635.

[4] B. D. Lindner, B. A. Coombs, M. Schaffroth, J. U. Engelhart, O. Tverskoy, F. Rominger, M. Hamburger, U. H. F. Bunz, *Org. Lett.* **2013**, *15*, 666-669.

[5] a) J. J. Bryant, Y. Zhang, B. D. Lindner, E. A. Davey, A. L. Appleton, X. Qian, U. H. F. Bunz, *J. Org. Chem.* **2012**, *77*, 7479-7486; b) S. Miao, S. M. Brombosz, P. v. R. Schleyer, J. I. Wu, S. Barlow, S. R. Marder, K. I. Hardcastle, U. H. F. Bunz, *J. Am. Chem. Soc.* **2008**, *130*, 7339-7344; c) A. L. Appleton, S. M. Brombosz, S. Barlow, J. S. Sears, J.-L. Bredas, S. R. Marder, U. H. F. Bunz, *Nat. Commun.* **2010**, *1*, 1-6; d) P. Biegger, S. Stolz, S. N. Intorp, Y. Zhang, J. U. Engelhart, F. Rominger, K. I. Hardcastle, U. Lemmer, X. Qian, M. Hamburger, U. H. F. Bunz, *J. Org. Chem.* **2015**, *80*, 582-589.

[6] A. L. Appleton, S. M. Brombosz, S. Barlow, J. S. Sears, J.-L. Bredas, S. R. Marder, U. H. F. Bunz, *Nat. Commun.* **2010**, *1*, 1-6.

[7] C. M. Cardona, W. Li, A. E. Kaifer, D. Stockdale, G. C. Bazan, *Adv. Mater.* **2011**, *23*, 2367-2371.
